# AUY922 induces retinal toxicity through attenuating TRPM1

**DOI:** 10.1186/s12929-021-00751-5

**Published:** 2021-07-23

**Authors:** Che-Hung Shen, Chi-Che Hsieh, Kuan-Ying Jiang, Chih-Yu Lin, Nai-Jung Chiang, Ting-Wei Li, Chun-Ting Yen, Wan-Ju Chen, Daw-Yang Hwang, Li-Tzong Chen

**Affiliations:** 1 National Institute of Cancer Research, National Health Research Institutes, No. 367, Sheng-Li Rd., North District, Tainan, 70456 Taiwan; 2 Department of Oncology, National Cheng Kung University Hospital, National Cheng Kung University, Tainan, 704 Taiwan; 3 Department of Life Sciences, National Cheng Kung University, Tainan, 704 Taiwan; 4 Institute of Molecular Medicine, National Cheng Kung University, Tainan, 704 Taiwan; 5 Department of Internal Medicine, Kaohsiung Medical University Hospital, Kaohsiung Medical University, Kaohsiung, 807 Taiwan; 6 Ph.D. Program in Tissue Engineering and Regenerative Medicine, Biotechnology Center, National Chung Hsing University, Taichung, 402 Taiwan; 7 Department of Ophthalmology, National Cheng Kung University Hospital, National Cheng Kung University, Tainan, 704 Taiwan

**Keywords:** Retinal toxicity, Photoreceptor, HSP90, CDC37, AUY922, TRPM1

## Abstract

**Background:**

Ocular adverse events are common dose-limiting toxicities in cancer patients treated with HSP90 inhibitors, such as AUY922; however, the pathology and molecular mechanisms that mediate AUY922-induced retinal toxicity remain undescribed.

**Methods:**

The impact of AUY922 on mouse retinas and cell lines was comprehensively investigated using isobaric tags for relative and absolute quantitation (iTRAQ)‑based proteomic profiling and pathway enrichment analysis, immunohistochemistry and immunofluorescence staining, terminal deoxynucleotidyl transferase dUTP nick end labeling (TUNEL) assay, MTT assay, colony formation assay, and western blot analysis. The effect of AUY922 on the Transient Receptor Potential cation channel subfamily M member 1 (TRPM1)-HSP90 chaperone complex was characterized by coimmunoprecipitation. TRPM1-regulated gene expression was analyzed by RNAseq analysis and gene set enrichment analysis (GSEA). The role of TRPM1 was assessed using both loss-of-function and gain-of-function approaches.

**Results:**

Here, we show that the treatment with AUY922 induced retinal damage and cell apoptosis, dysregulated the photoreceptor and retinal pigment epithelium (RPE) layers, and reduced TRPM1 expression. Proteomic profiling and functional annotation of differentially expressed proteins reveals that those related to stress responses, protein folding processes, regulation of apoptosis, cell cycle and growth, reactive oxygen species (ROS) response, cell junction assembly and adhesion regulation, and proton transmembrane transport were significantly enriched in AUY922-treated cells. We found that AUY922 triggered caspase-3-dependent cell apoptosis, increased ROS production and inhibited cell growth. We determined that TRPM1 is a bona fide HSP90 client and characterized that AUY922 may reduce TRPM1 expression by disrupting the CDC37-HSP90 chaperone complex. Additionally, GSEA revealed that TRPM1-regulated genes were associated with retinal morphogenesis in camera-type eyes and the JAK-STAT cascade. Finally, gain-of-function and loss-of-function analyses validated the finding that TRPM1 mediated the cell apoptosis, ROS production and growth inhibition induced by AUY922.

**Conclusions:**

Our study demonstrates the pathology of AUY922-induced retinal toxicity in vivo. TRPM1 is an HSP90 client, regulates photoreceptor morphology and function, and mediates AUY922-induced cytotoxicity.

**Supplementary Information:**

The online version contains supplementary material available at 10.1186/s12929-021-00751-5.

## Background

Heat shock protein 90 (HSP90), an ATP-dependent molecular chaperone, governs the folding and maturation, intracellular disposition, and proteolytic turnover of numerous client proteins [1]. By hydrolyzing ATP, HSP90 collaborates with other co-chaperones such as heat shock protein 70 (HSP70) and cell division cycle 37 (CDC37) to facilitate protein folding, which is a mechanism to regulate the conformation and activity of clients [2–4]. HSP90 clients include numerous cancer-related proteins such as membrane receptors (i.e., EGFR and HER2) and signal-transduction proteins (i.e., BRAF and AKT) [5]. Targeting HSP90, therefore, is a potential therapeutic strategy for cancer treatment. In fact, many small molecules have been designed to block ATP binding to HSP90 and have shown anti-tumor activity [2, 6, 7]. AUY922, a potent HSP90 inhibitor, shows activity against several types of human cancer cell as a single agent or in combination with other therapeutics [8–15]. Phase II clinical studies show that AUY922 is well tolerated and active in patients with HER2-positive metastatic breast cancer who progressed on trastuzumab-based therapy [16]; it is also effective in patents with stage IV NSCLC, particularly in patients with ALK rearrangements and EGFR mutations [17]. However, approximal 80% of patents reported ocular adverse events (AEs). Such vision-related complications, especially night blindness, limit the duration and applicable dosage of AUY922 and may be responsible for partial or modest responses in patients with chemotherapy refractory advanced pancreatic cancer [18], EGFR-mutant lung cancer [19], and heavily pretreated gastrointestinal stromal tumors [20]. Recently, TAS-116, a novel HSP inhibitor, has been shown to have potent anti-tumor activity with reduced ocular toxicity in preclinical animal models, compared to AUY922 [21]. However, night blindness remains the main ocular AE in approximately 30% of patients [22]. This indicates that visual impairment is a significant obstacle in the clinical application of HSP90 inhibitors, and existing assessments in preclinical animal models are not sufficient to model HSP90 inhibitor-induced retinal disorder. It is important to delineate the pathology and molecular mechanisms of HSP90i-induced retinal toxicity.

TRPM1 (originally named melastatin 1) belongs to the transient receptor potential (TRP) superfamily and is mainly expressed in pigmentated cells of the eye and skin [23]. It is a constitutively open Ca^2+^ entry channel and has been proposed to be involved in synapse formation and signal mediation from photoreceptors to ON bipolar cells [24]. In response to light stimulation, depolarized photoreceptors release glutamates into the synapse, and glutamates binds to and activates metabotropic glutamate receptor 6 (mGluR6) at the dendritic tips of ON bipolar cells. Activated mGluR6 closes the TRPM1 Ca^2+^ channel, which in turn results in rod bipolar terminal contraction and abrogates neurotransmission [24–26]. TRPM1 knockout mice exhibit no b-wave in electroretinogram (ERG) and completely lack photoresponses [25]. Genetically, mutations in TRPM1 are associated with congenital stationary night blindness syndrome (CSNB) and coat color phenotypes in Appaloosa horses [27–29]. Potential pathogenic mutations of TRPM1 have also been identified in patients with CSNB. These loss-of-function TRPM1 mutations are inherited in an autosomal recessive pattern and associated with CSNB syndrome [30, 31]. These findings strongly support that TRPM1 is critical for vision.

Collectively, the CSNB phenotype described in patients and animals, the consequences of loss of TRPM1 identified in mouse models, and the biological functions discovered in retina support that TRPM1 is a key mediator of AUY922-induced retinal toxicity. We hypothesized that TRPM1 could play a critical role in AUY922-induced retinal dysfunction. In this study, we report ocular AEs and abnormal ERGs in patients who received AUY922. In vivo mouse models, AUY922 administration clearly induced damage and dysregulation of retinas and reduced TRPM1 expression. Proteomic profiling and functional annotation of differentially expressed proteins reveals that those related to stress responses, protein folding processes, regulation of apoptosis, cell cycle and growth, reactive oxygen species (ROS) response, cell junction assembly and adhesion regulation, and proton transmembrane transport were significantly enriched in AUY922-treated cells. In vitro cell models, AUY922 triggered caspase-3-dependent apoptosis, increased ROS production and inhibited cell growth. We determined TRPM1 as a bona fide HSP90 client and characterized that AUY922 may reduce TRPM1 expression by disrupting the CDC37-HSP90 chaperone complex. We identified that TRPM1-regulated genes were associated with retinal morphogenesis in camera-type eyes and the JAK-STAT cascade. TRPM1 plays a role in regulating intracellular Ca^2+^ concentrations and might be involved in cilia formation and protection of photoreceptors from ROS stress. Moreover, we characterize that the two TRPM1 mutations in the pore loop of the TRPM1 channel—(c.3070A > T, p. I1024F and c.3206G > A, p. C1069Y) that were identified in patients with CSNB are functionally defective mutations. We validated that TRPM1 was critical for photoreceptor function and maintenance, and that TRPM1 mediated AUY922-induced apoptosis, ROS production and growth inhibition. Our present work revealed the histopathology and molecular mechanisms underlying AUY922-induced retinal toxicity through attenuation of TRPM1 expression and provides new insights into the biological functions of TRPM1 in photoreceptors.

## Methods

### Patients

The phase II investigator-initiated trial of AUY922, a heat shock protein inhibitor in patients with metastatic gastrointestinal stromal tumors (GISTs) who failed to response to imatinib and sunitinib therapy (NCT01389583) was conducted in several Taiwan Cooperative Oncology Group (TCOG)-affiliated medical centers between October 2011 and January 2015. All patients gave signed informed consent and received weekly AUY922 treatment at 70 mg/m^2^.

### Cell culture, transfection, and retroviral and lentiviral infection

293 cells (a permanent line established from primary embryonic human kidney, which was transformed with sheared human adenovirus type 5 DNA), A375 and Mel1617 cells (human metastatic melanoma cell lines) were obtained from Dr. Bin Zheng [32]. 661 W cells (an immortalized cone photoreceptor cell line derived from the retinal tumor of a mouse expressing SV40 T antigen) [33] and ARPE19 cells (a spontaneously arising human RPE cell line) [34] were purchased from ATCC. A375 cells were cultured in RPMI1640 (HyClone), 293, Mel1617, and 661 W cells were maintained in DMEM (HyClone), and ARPE19 cells were kept in DMEM/F-12 (Gibco) containing 10% fetal bovine serum (FBS; Hyclone) and 100U/ml penicillin/streptomycin (Gibco). All cell lines tested negatively for mycoplasma with the MycoSensor PCR Assay Kit (Agilent Technologies).

Transfection, retroviral and lentiviral infection were performed as previously described [32]. Briefly, 293 cells were polyethylenimine (PEI) transfected with Ampho packaging vector and pBabe-Puro retroviral vector encoding the gene of interest to produce retroviruses, or with packaging plasmids encoding VSV-G, gag-pol, Rev, and pLKO lentiviral vector encoding shRNA against TRPM1 or CDC37 to produce lentiviruses. Culture supernatants containing virus were collected and filtered 48 h post-transfection to infect cultured cells in the presence of 4 mg/ml polybrene (Sigma-Aldrich). When indicated, stable populations were obtained and maintained by selection with puromycin (Sigma-Aldrich).

### Constructs

3xFLAG tag was added on the N-terminal of full‐length human *TRPM1* cDNA and was cloned into pBABE-Puro vector using PCR-based subcloning. *TRPM1* mutants were generated using PCR-based mutagenesis. pLKO constructs containing shRNAs against human *TRPM1* (shTRPM1#74: TRCN0000043973; shTRPM1#21: TRCN0000429621), mouse *Trpm1* (shTRPM1#7: TRCN0000070007); and *CDC37* (shCDC37#32: TRCN0000116632; shCDC37#33: TRCN0000116633), which were purchased from the National RNAi Core Facility at Academia Sinica (Taipei, Taiwan). All constructs were verified by sequencing.

### Materials

Anti-human TRPM1 (F-3, western blotting 1: 250; immunoprecipitation 1:50; immunohistochemistry 1:100), anti-HSP70 (W27, for western blotting, 1:1000), anti-HSP90 α/β (F-8, for western blotting, 1:1000), anti-CDC37 (C-11, for western blotting, 1:1000) antibodies, and anti-HSP90 α/β conjugated agarose were from Santa Cruz Biotechnology. Anti-mouse Trpm1 (western blotting 1:200; immunohistochemistry 1:100; immunofluorescence 1:100) was from Novus Biologicals. Anti-cleaved caspase 3 (Asp175) (western blotting 1:1000; immunohistochemistry 1:100), anti-AKT (western blotting 1:1000; immunohistochemistry 1:100), anti-phospho-AKT(Ser473) (D9E, immunohistochemistry 1:100), anti-β-Actin (13E5, western blotting 1:2000; immunofluorescence 1:500), anti-PARP1 (# 9542, western blotting 1:1000) and anti-Ki67 (D2H10, immunohistochemistry 1:100) antibodies were purchased from Cell Signaling Technology. Anti-Rhodopsin (Rho 1D4, immunohistochemistry 1:100), anti-RPE65 (immunofluorescence 1:250), anti-FLAG M2 (western blotting 1:1000) antibodies and anti-FLAG M2 affinity agarose gel were from Sigma-Aldrich. Anti-GFAP antibody (immunohistochemistry 1:100) was from Dako. Biotinylated Peanut Agglutinin ((immunohistochemistry 1:500) was obtained from Vector Laboratories. AUY922 and MG132 were from MedChem Express.

### Western blotting and co-immunoprecipitation

Western blotting and immunoprecipitation were performed as previously described [32]. Briefly, cell lysates were prepared using lysis buffer containing Tris pH 7.4, 150 mM NaCl, 1% NP-40, 1 mM EDTA, 50 mM NaF, 10 mM β-glycerophosphate, 10 nM calyculin A, 1 mM Na_3_VO_4_ and protease inhibitors, and normalized by protein concentrations using the Bradford method (Bio-Rad). For western blotting, cell lysates were boiled in Laemmli sample buffer and separated on 8%–12% SDS-PAGE and transferred to Immobilon-P PVDF Membrane (Sigma-Aldrich). The membranes were blocked in TBST containing 5% nonfat milk, incubated with primary antibodies based on the manufacturer’s instructions, followed by incubation with horseradish peroxidase-conjugated goat anti-rabbit or anti-mouse IgG (Thermo Fisher) and enhanced chemiluminescence detection (Sigma-Aldrich).

For co-immunoprecipitation, cell lysates were incubated with primary antibodies, anti-FLAG M2 affinity agarose gel, or anit-HSP90 conjugated agarose at 4 °C overnight, followed by incubation with protein A/G Sepharose for an additional 1 h at 4 °C, when applicable. Beads were washed three times with lysis buffer and boiled in Laemmli sample buffer, and immune complexes were analyzed by SDS-PAGE and western blotting.

### MTT assay and colony formation assay

MTT and cell clonogenic growth assays were performed as previously described [32]. Briefly, cells were seeded in 96-well plates, and variant concentrations of AUY922 were added the following day. After a 72 h incubation, cell viability was examined using a CellTiter 96 AQueous Assay kit based on the manufacturer’s instructions (Promega). Combined MTS [3-[4, 5-dimethyliazol-2-yl]-5-(3-carboxymethoxyphenyl)-2-(4-sulfophenyl)-2H-tetrazolium, inner salt] and PMS [phenazine methosulfate] solution was added into each well of cell-containing 96-well plate for 2 – 3 h at 37 °C. The amount of soluble formazan was measured based on the changes in absorbance at 490 nm using an ELISA plate reader (Multiskan FC, Thermo Fisher Scientific).

For colony formation assay, cells were seeded in 6-well plates at a low density. The colonies were stained with crystal violet after 10–14 d. For AUY922 treatment, cells were replaced with AUY922 containing fresh media every 2–3 d.

### Cytosolic Ca^2+^ concentration measurement

For cytosolic Ca^2+^ measurement, cells were washed in PBS, trypsinized, and then neutralized with culture medium. After two washes with PBS, cells were collected by centrifugation and resuspended in PBS 5 μM Fluo-8 AM (Santa Cruz) for 30 min at 37 °C in the dark. After washing in PBS, fluorescence was measured with flow cytometry (excitation at 488 nm and emission at 515–545 nm, Attune NxT, Life Technologies). The mean fluorescence intensity (MFI) for 10,000 cells/sample was determined using FlowJo software (TreeStar) and plotted using Prism 8 (GraphPad Software).

### Apoptosis analysis

Apoptotic cells were detected by using FITC annexin V apoptosis detection kit (BD Biosciences) according to the manufacturer’s protocol. Briefly, cells were washed in PBS, trypsinized, and then neutralized with culture medium. After two washes with PBS and further centrifugation, 1 × 10^5^ cells were resuspended in 100 μl Annexin binding buffer containing 1 μl of 100 μg/ml PI working solution and 5 μl Annexin V FITC-conjugated, and incubated for 15 min at 4 °C in the dark. Each sample was added with Annexin binding buffer to reach 500 μl. Samples were analyzed by flow cytometry (Attune NxT, Life Technologies). Cellular distribution depending on Annexin V and PI positivity allowed the measure of the percentage of viable cells (Annexin V and PI negative cells), early apoptosis (Annexin V-positive and PI negative cells), late apoptosis (Annexin V and PI-positive cells), and necrosis (Annexin V-negative and PI-positive cells). The data were analyzed using FlowJo software (TreeStar) and plotted using Prism 8 (GraphPad Software).

### ROS production assay

Intracellular ROS levels were measured with cell permeant reagent 2’,7’-dichlorofluorescein diacetate (DCFDA, Abcam) based on the manufacturer’s instructions. Briefly, 661 W cells were treated with indicated concentrations of AUY922 for 48 h. Cells were washed in PBS, trypsinized, and then neutralized with culture medium. After two washes with PBS and further centrifugation, cells were resuspended in culture medium containing 20 µM DCFDA and incubated for 30 min in the dark at 37 °C. The fluorescence was measured with flow cytometry (excitation at 488 nm and emission at 515–545 nm, Attune NxT, Life Technologies). The MFI for 10,000 cells/sample was determined using FlowJo software (TreeStar) and plotted using Prism 8 (GraphPad Software). 55 µM Tert-butyl hydroperoxide (TBHP) were served as positive control.

### Reverse-transcription and real-time qPCR

RNA samples were isolated using the RNeasy Mini kit (Qiagen) and reverse transcribed (~ 2 mg) using the High-Capacity cDNA Reverse Transcription Kit (Applied Biosystems). qPCRs were performed using Fast SYBR Green Master Mix (Applied Biosystems) on a 7500 Fast Real-Time PCR System (Applied Biosystems). Each sample was tested in triplicates, and results were normalized with the expression of the housekeeping *β-Actin* gene. Specific primer sequences used in this study were as follows:

*Lrp5* forward, 5’-CCTCACCATTGATTATGCCGACC-3’; *Lrp5* reverse, 5’- GATCGTCAGCTATCACCATGCG-3’; *Hes1* forward, 5’- GGAAATGACTGTGAAGCACCTCC-3’; *Arl13b* forward, 5’-TCAGGAAAGCCTATATTGGTGCT-3’; *Hes1* reverse, 5’- GAAGCGGGTCACCTCGTTCATG-3’; *Arl13b* reverse, 5’-AGGCACTTGTGCTCGTTGAC-3’; *Opn1sw* forward, 5’-CAGCCTTCATGGGATTTGTCT-3’; *Opn1sw* reverse, 5’- CAAAGAGGAAGTATCCGTGACAG -3’; *Nrf2* forward, 5’-CTTTAGTCAGCGACAGAAGGAC-3’; *Nrf2* reverse, 5’-AGGCATCTTGTTTGGGAATGTG-3’; *Ift88* forward, 5’-TGAGGACGACCTTTACTCTGG-3’; *Ift88* reverse, 5’-CTGCCATGACTGGTTCTCACT-3’; *Cep164* forward, 5’-AGAGTGACAACCAGAGTGTCC-3’; *Cep164* reverse, 5’-GGAGACTCCTCGTACTCAAAGTT-3’; *Rpgrip1l* forward, 5’-GCCGGTGAAAGATACAGGTCT-3’; *Rpgrip1l* reverse, 5’-ACGCAAAAATCTGTCTTCCAGT-3’; *β-Actin* forward, 5’-GCTACAGCTTCACCACCACA-3’; *β-Actin* reverse, 5’-TCTCCAGGGAGGAAGAGGAT-3’.

### In vivo xenograft studies

All animal studies were approved by the Institutional Animal Care and Usage Committee (IACUC) of the National Health Research Institutes (NHRI, Taiwan). Four- to six-week old female Nu/Nu nude mice (LASCO, Taipei, Taiwan) were housed in a specific pathogen-free environment in the animal facility of NHRI. A375 cells (6 × 10^6^ cells/mouse) were mixed with Matrigel (BD Biosciences) and subcutaneously inoculated into the flanks of the mice. Tumor growth was monitored, and tumor size was calculated using the following equation: [length × (width)^2^]/2. When the tumor size reached approximately 100 mm^3^, the mice were intraperitoneally injected with sunflower oil alone (Mock, *n* = 6) or AUY922. AUY922 (25 mg/kg) was dissolved in DMSO, diluted in sunflower oil, and injected three times per week (3qw, *n* = 6) or five times per week (5qw, *n* = 6). The mice were euthanized when the tumor size reached 1000 mm^3^.

### Histopathologic analysis of mouse tissues

Eyes and xenograft tumors were collected 24 h after the final dose, fixed in 4% paraformaldehyde for 1 h, transferred to 10% (v/v) buffered formalin solution for overnight fixation, and stored in 70% ethanol until processed for routine paraffin embedding. The paraformaldehyde-fixed eyecups and tumors were dissected to obtain sclerochoroidal flat mounts, which were then transferred to formalin solution and embedded in paraffin. Four micrometer-thick sections were prepared and subjected to antigen retrieval in antigen unmasking buffer pH9.0 (Vector Laboratories) at 95 °C for 30 min and blocked with PBS containing 5% FBS and 0.5% Triton for 30 min. The sections were incubated with primary antibodies against TRPM1, AKT, phospho-AKT, cleaved caspase-3 (Asp175), rhodopsin, Ki67, or GFAP at 4 °C overnight. Subsequently, sections were stained using Dako Real Envision HRP/DAB detection reagent (Dako) according to the manufacturer’s instructions. Peanut agglutinin lectin (PNA) labeling was performed using biotinylated PNA (Vector Laboratories), stained with streptavidin peroxidase (1:500, Vector Laboratories) and visualized using DAB solution (Dako). All sections were counterstained with hematoxylin (Muto Pure Chemicals) and mounted with Malinol medium (Muto Pure Chemicals). The stained tissues were viewed and photographed using a light microscope (Leica DM2000). The gain was set to 1.0x, saturation was set to 1.00, and gamma was set to 1.01 for imaging.

Cleaved caspase-3-positive cells were identified by brown stains in the cytoplasm using NIH ImageJ software. The relative immunoreactivities for GFAP were scored with a 4-point grading system: 0, negligible staining; 1, Müller cell end feet region plus a few proximal processes; 2, some Müller cell end feet extending to the ONL; and 3, Müller cell end feet plus many dark processes from the GCL to the outer margin of the ONL [35, 36]. The quantification of the cleaved caspase3-positive cells and the scoring of GFAP expression in retinas (*n* = 4) were conducted in a blinded fashion by two independent investigators and plotted using Prism 8 (GraphPad Software).

For TUNEL assay, rehydrated sections were stained with a TUNEL Assay Kit-HRP-DAB (Abcam) according to the manufacturer’s instructions. Briefly, the sections were treated with Proteinase K in PBS for 10 min at room temperature, followed by endogenous peroxidase quenching. The sections were then incubated with terminal deoxynucleotidyl transferase (TdT) reaction buffer for 10 min, followed by incubation with TdT reaction mix for 1 h at 37 °C and rinsing with stop buffer for 10 min. The reaction products were visualized using DAB solution, counterstained with Methyl green and mounted with Malinol medium (Muto Pure Chemicals). For RPE flat mount sections, hematoxylin was used for counterstaining. The stained tissues were photographed under a light microscope. TUNEL-positive cells were identified by brown staining of the nucleus using NIH ImageJ software. The quantification of TUNEL analysis in retinas (*n* = 4) was conducted in a blinded fashion by two independent investigators and plotted using Prism 8 (GraphPad Software).

For immunofluorescence staining, the sections from sclerochoroidal flat mounts were immunolabeled with primary antibodies against Trpm1, RPE65, or β-Actin at 4 °C overnight, and then incubated with secondary antibodies: Alexa Fluor 488- or 594-conjugated anti-rabbit or mouse IgG (Thermo Fisher Scientific) in the dark. The sections were then counterstained with Hoechst 33342 (Thermo Fisher Scientific) and mounted with fluorescence mounting medium (Dako). Fluorescence microscopy was performed on a NIKON TE2000EPS-C1-S1 microscope. The fluorophores were excited as follows: Hoechst 33342 at 408 nm, Alexa Fluor 488 at 488 nm, and Alexa Fluor 594 at 543 nm. Hoechst 33342 emission was collected at 450 ± 35 nm, Alexa Fluor 488 at 515 ± 30 nm, and Alexa Fluor 594 at 590 ± 50 nm. For analyses of fluorescence intensity, microscope setting parameters were kept constant and imaged on the same day. Images were taken from a minimum of four stained tissues by using 40 × objective lenses (NIKON Plan Apo 40X N.A. 0.95 DIC M/N2 0.11–0.23 WD. 0.14 mm) and analyzed using the Nikon EZ-C1 software.

### RNA sequencing and gene set enrichment analysis (GSEA)

RNA-seq libraries were prepared using the KAPA mRNA HyperPrep Kit (KAPA Biosystems, Roche, Basel, Switzerland) and validated using the Qsep 100 DNA/RNA Analyzer (BiOptic Inc., Taiwan). Libraries were sequenced on a NovaSeq 6000 sequencer (Illumina, CA, USA). Clean reads were aligned to the mouse genome (GRCm38) using HISAT2 (version 2.1.0) after removing low-quality reads. The differential expression of genes between TRPM1-overexpressing and control cells were computed using the fragments per kilobase of transcript per million mapped reads calculated by featureCounts (version 2.0.0). Raw read counts were imported into edgeR (version 3.28.1) and analyzed using the R package of DESeq (version 1.40.0). Genes with a false discovery rate (FDR) P-value < 0.05 adjusted by the Benjamini–Hochberg (BH) method were considered differentially expressed genes (DEGs). Gene set enrichment analysis of the genes differentially expressed upon TRPM1 overexpression was performed using the Gene Set Knowledgebase (GSKB) hallmark gene sets. The RNAseq data can be accessed in the Gene Expression Omnibus database under accession number GSE165691.

### Isobaric tags for relative and absolute quantitation (iTRAQ)‑based proteomic profiling and pathway enrichment analysis

Protein sample was prepared as follows: 661 W cells treated with DMSO or 30 nM AUY922 were lysed with lysis buffer (20 mM HEPES buffer, 0.1% SDS, 1 mM EDTA, 1 mM phenylmethylsulfonyl fluoride), sonicated in Sonicator 3000 (Misonix) with a rotating device and a horn (1 min; output level of 1; 3 s ON and 10 s OFF) and centrifuged. The protein concentration of the lysates was determined with a BCA assay (Thermo Fisher Scientific). The lysates were diluted in 200 mM triethylammonium bicarbonate (Sigma-Aldrich), reduced with 5 mM tris-(2 carboxyethyl)-phosphine (Sigma-Aldrich) at 60 °C for 45 min, followed by cysteine blocking with 10 mM methyl methanethiosulfonate (Sigma-Aldrich) at 25 °C for 30 min and digestion with trypsin at 37 °C for 16 h. The DMSO- and AUY922-treated peptides were labeled with iTRAQ reagents 114 and 116, respectively. The labeled peptides of each group were pooled and dried by vacuum centrifugation.

LC–MS/MS analysis was performed as follows: the dried peptide mixtures were reconstituted in 0.1% formic acid, desalted through a reverse-phase column (Zorbax 300SB-C18, 0.3 × 5 mm; Agilent Technologies), and separated on a column (Waters BEH 1.7 µm, 100 µm I.D. × 10 cm with a 15 µm tip) with a multi-step gradient of 99.9% acetonitrile/0.1% formic acid at a flow rate of 0.3 μl/min for 2 h. Full-scan MS was performed using a 2D linear ion trap mass spectrometer (Orbitrap Elite, Thermo Fisher Scientific) with Xcalibur 2.2 software (Thermo Fisher Scientific) over a range of 400 to 2000 Da and a resolution of 120,000 at m/z 400. The 16 data-dependent MS/MS scan events (8 CID, 8 HCD) were followed by one MS scan for the 8 most abundant precursor ions in the preview MS scan. The m/z values selected for MS/MS were dynamically excluded for 80 s with a relative mass window of 15 ppm. The electrospray voltage was set to 2.0 kV, and the temperature of the capillary was set to 200 ℃. MS and MS/MS automatic gain control were set to 1,000 ms (full scan) and 300 ms (MS/MS) or 3 × 10^6^ ions (full scan) and 3 × 10^4^ ions (MS/MS) for maximum accumulated time or ions, respectively.

Protein identification and iTRAQ quantification were performed with Proteome Discoverer software (version 1.4, Thermo Fisher Scientific) with the Mascot search engine (Matrix Science, version 2.5) using the SwissProt database. A mass tolerance of 10 ppm was permitted for intact peptide masses, and 0.2 Da for HCD/0.5 Da for CID fragment ions with allowance for two missed cleavages made from trypsin digestion, oxidized methionine, acetyl (protein N-terminal), and iTRAQ4plex (peptide N-terminal, K) as variable modifications and methylthio (cysteine) as static modification. Peptide-spectrum matches (PSMs) were then filtered based on high confidence and Mascot search engine rank 1 of peptide identification to ensure an overall false discovery rate below 0.01. Proteins with single peptide hit were removed. The quantitative protein data were exported from Proteome Discoverer using integration methods of the most confident centroid with 20 ppm tolerance. The quantity of identified peptides was calculated and compared between the AUY922- and DMSO-treated groups using iTRAQ reported ion intensities. The mean value of the unique peptide of each protein was used to calculate the protein iTRAQ ratio. A 1.2-fold change of iTRAQ ratios (ratio < 0.833 or > 1.2) was selected as the cutoff for differentially expressed protein. Proteins with iTRAQ ratios below the low range (0.833) were considered to be downregulated, whereas those above the high range (1.2) were considered to be upregulated proteins.

For bioinformatic analysis and annotation, we performed functional enrichment analyses of the differentially expressed proteins using Metascape [37]. The related gene ontology (GO) and KEGG pathway annotations were ranked based on their − log10 (q-value), and we selected those with q-value < 0.01.

### Statistical analysis

Statistical analyses were performed using Prism 8 (GraphPad Software). The in vitro experiments were performed in biological triplicates each time and independently repeated at least 3 times. Data are presented as the mean ± s.e.m. and the number (*n*) of samples is indicated. Student’s t-test (two-tailed) was used to compare differences between the control and experimental groups. For all statistical analyses, differences were labeled as *, P < 0.05; **, P < 0.01; *** P < 0.001; or ****, P < 0.0001. P values < 0.05 were considered statistically significant. n.s. = not significant.

## Results

### AUY922 induces retinal toxicity in GIST patients

**Fig. 1:**
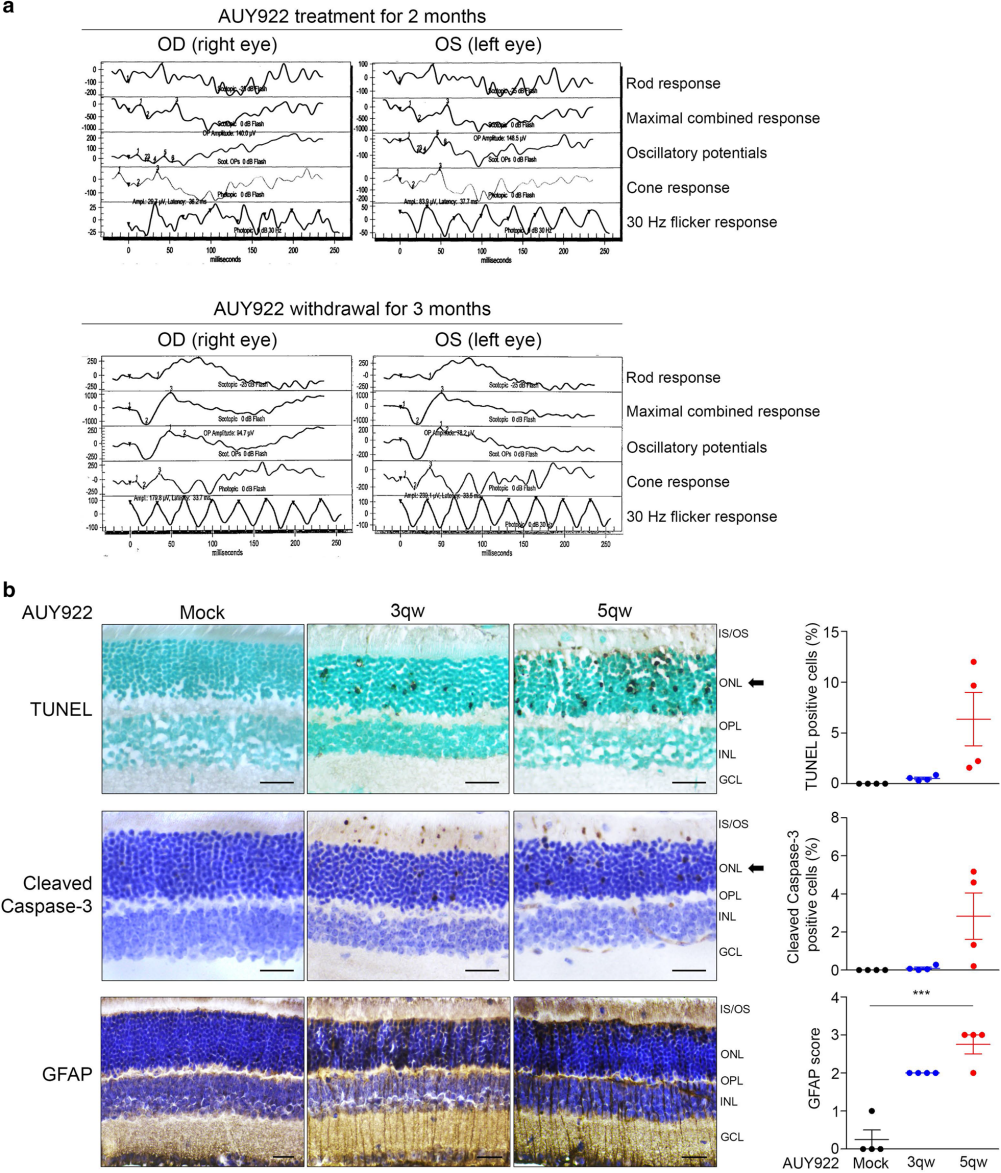
Treatment with AUY922 induced retinal dysfunction. **a** Full field electroretinogram (ffERG) of a patient with refractory gastrointestinal stromal tumor who received 70 mg/m^2^ weekly AUY922 treatment for 2 months before withdrawing treatment for 3 months. OD: right eye, OS: left eye. **b** Representative images of apoptotic cells (top), cleaved caspase-3 (middle) and GFAP (bottom) expression, as determined by TUNEL assay and IHC analysis, in mouse eye samples from nude mice bearing A375 xenograft tumors, after treatment with mock, 25 mg/kg AUY922 three times weekly (3qw), or five times weekly (5qw) for 2 w. IS: inner segment; OS: outer segment; ONL: outer nuclear layer; OPL: outer plexiform layer; INL: inner nuclear layer; GCL: ganglion cell layer. Scale bar: 25 μm. The arrow indicates the layer vulnerable to AUY922. The percentage of TUNEL-positive and cleaved caspase-3-positive cells and the GFAP IHC score are presented on the right. Data are presented as the mean ± s.e.m. The P values were determined by two-tailed Student’s t-test, *** P < 0.001. *n* = 4

In our phase II trial of AUY922 monotherapy as a later-line treatment (NCT01389583), a total of 25 patients with metastatic GISTs who failed to respond to imatinib and sunitinib therapy were enrolled. The common all-grade AEs were fatigue (78%), ocular toxicities (74%), anemia (56%) and diarrhea (55%). Multiple vision-related symptoms were reported, including all grades of night blindness (74%), blurred vision (70%) and flashing light (59%). One patient had grade III night blindness (4%), and two had grade III blurred vision (7%). Sixty-two percent of patients required dose modifications or treatment interruption due to ocular toxicities [20]. Routine ophthalmological examinations, including visual acuity, fundus imaging under the slit lamp and visual field, were unremarkable after 2 months of AUY922-treatment. However, the full-field ERG (ffERG) revealed significantly abnormal waveforms in all modalities, which recovered 3 months after drug discontinuation (Fig. 1a and Additional file 1: Figure S1a). Notably, the abnormal b-wave was observed in both rod and cone responses, and the reduced amplitude was observed in the 30 Hz flicker response, indicating the impairment of photopic and scotopic vision in response to white light stimulation. The results of ffERGs indicate that treatment with AUY922 induces reversible retinal dysfunction, which was the main cause for dosing schedule modification, reduction or interruption and limited the clinical efficacies of AUY922.

### Treatment with AUY922 attenuates TRPM1 expression and induces retinal injury

Next, we investigated the effects of AUY922 on mouse retinas. The treatment doses of AUY922 were determined by its tumor suppression efficacy in a human melanoma xenograft mouse model. A375 cells were grown as xenograft tumors in nude mice and mice were treated with intraperitoneal injection of mock alone, AUY922 (25 mg/kg/3qw), or AUY922 (25 mg/kg/5qw) for 2 weeks. Mice treated with AUY922 both 3qw and 5qw showed a significant reduction in tumor growth compared to mock-treated animals over 2 weeks of the treatment period (Additional file 1: Figure S1b). The body weights of animals treated with AUY922 were comparable to those of mock-treated animals. Eyes from mock- and AUY922-treated tumor-bearing mice were collected and subjected to histochemical analysis. We did not observe significant differences in the thickness of the ounter nuclear layer (ONL), consisting of cell bodies of rod and cone photoreceptor cells or the inner nuclear layer (INL), consisting mainly of the cell bodies of horizontal, bipolar, and amacrine cells between mock- and AUY922-treated retinas (Additional file 1: Figure S1d and e). Next, we assessed cell apoptosis and the expression of glial fibrillary acidic protein (GFAP), which reflects nonspecific responses to retinal diseases and injuries [38]. Terminal dUTP nick end labeling (TUNEL), cleaved caspase-3 and GFAP immunohistochemistry (IHC) assays were performed on serial sections from eyes treated with mock alone or AUY922. We found that AUY922 (5qw) markedly increased the numbers of TUNEL-positive cells (6.4 ± 2.6%, *n* = 4) and cleaved caspase-3-positive cells positive cells (2.8 ± 1.2%, *n* = 4) in the ONL. The IHC analysis of GFAP were scored using 4-point grading system [35, 36] and showed that treatment with AUY922 induced the expression of GFAP (mock: 0.25 ± 0.25, AUY922 (3qw): 2 ± 0 and AUY922 (5qw): 2.75 ± 0.25 arbitrary units, *n* = 4) (Fig. 1b and Additional file 1: Figure S1f). These suggest that AUY922 triggers photoreceptor cell apoptosis and induces retinal injury responses.

**Fig. 2:**
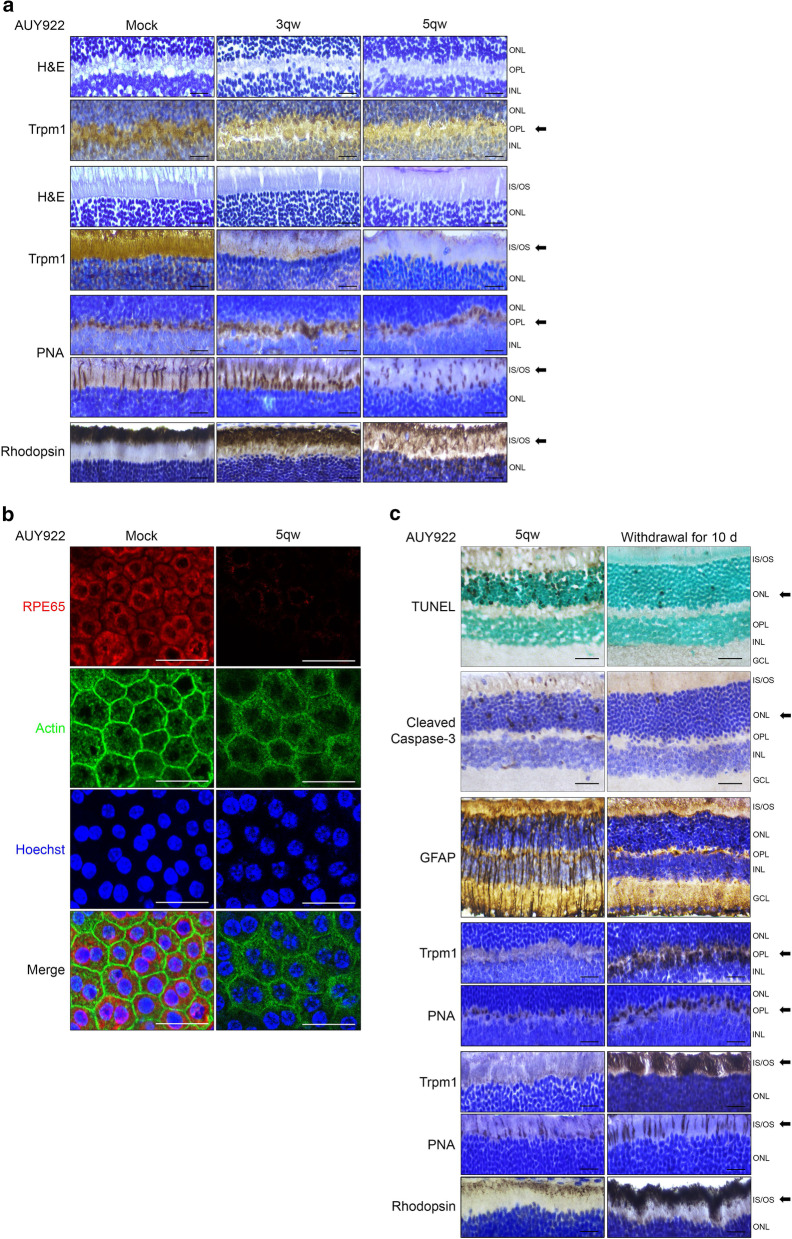
Treatment with AUY922 attenuated TRPM1 expression and dysregulated photoreceptors and RPE cells in the mouse retina. **a** Representative images of H&E stain and IHC analysis for TRPM1, PNA and rhodopsin expression in mouse eye samples from the AUY922 treatment experiment in Fig. 1b. Scale bar: 25 μm. *n* = 4. **b** Representative images of RPE65 and Actin expression, as determined by fluorescence immunohistochemistry and confocal scanning laser microscopy, in mouse RPE flat mounts from the AUY922 treatment experiment in Fig. 1b. Scale bar: 25 μm. *n* = 4. **c** Representative images of TUNEL assay and IHC analysis for cleaved caspase-3, GFAP, Trpm1, PNA and rhodopsin expression in mouse eye samples from mice treated with 25 mg/kg AUY922 5qw for 2 w before drug withdrawal for 10 d. Scale bar: 25 μm. *n* = 4. IS: inner segment; OS: outer segment; ONL: outer nuclear layer; OPL: outer plexiform layer; INL: inner nuclear layer. GCL: ganglion cell layer. The arrow indicates the AUY922-sensitive layer

TRPM1 is critical for neurotransmission and visual performance, and the loss of TRPM1 is associated with loss of b-wave in ERGs in SCNB patients. In our clinical observations, the patients who received AUY922 presented abnormal ERGs without b-waves. We therefore hypothesized that TRPM1 could be involved in mediating AUY922-induced retinal toxicity and visual impairment. IHC analysis showed that TRPM1 was expressed in the ONL and INL and enriched in the outer plexiform layer (OPL), the terminals of the photoreceptor synapse with dendritic processes of bipolar cells and the junction between the photoreceptor's inner and outer segments (IS/OS) (Fig. 2a). Treatment withAUY922 reduced TRPM1 expression in a dose-dependent manner, especially in the OPL and IS/OS layers, where neurotransmission and phototransduction take place, respectively (Fig. 2a).

### AUY922 dysregulates photoreceptor and retinal pigment epithelium (RPE) cells

We next examined the effect of AUY922 on the integrity of synapses (OPL) and the segmentation of photoreceptor cells (IS/OS). The retinal sections were stained with rhodamine-labeled peanut agglutinin (PNA) to assess the integrity of cone photoreceptor synapses and the morphology of the segment fragments of cone photoreceptor cells [39]. Upon AUY922 administration, PNA patterns did not change substantially in the OPL but they became irregular in the IS/OS layer (Fig. 2a). We found that treatment with AUY922 reduced the number of cone photoreceptor cells with shortened outer segment fragments, and also decreased the rhodopsin-stained rod outer segment fragments with an abnormal pattern (Fig. 2a), indicating that AUY922 damages the segment fragments of photoreceptor cells. Phagocytosis and the retinoid cycle of retinal pigment epithelium (RPE) cells are required for lifelong maintenance and renewal of outer segment fragments [40]. To examine whether AUY922 affects the function of RPE cells, phagocytosis was assessed by F-Actin morphology, and the retinoid cycle by the expression of RPE65, an RPE-specific enzyme that catalyzes the retinoid cycle to regenerate 11-cis-retinal [41, 42]. As shown in Fig. 2b, the RPE flat mounts from AUY922-treated retinas lost their contiguous lateral circumferential F-Actin and RPE65 expression was significantly reduced. However, we did not detect apoptotic RPE cells in AUY922-treated retinas (Additional file 2: Figure S2). These findings suggest that treatment with AUY922 could reduce the phagocytosis and retinoid cycle by RPE cells, resulting in impairment of lifelong maintenance and renewal of photoreceptor segment fragments. In patients, normal b-waves returned by 3 months after drug withdrawal. We next examined whether the retinas could recover after discontinuing AUY922 in mice. We assessed the mouse retinas 10 d after drug withdrawal and found that retinal injury was ameliorated, fewer apoptotic and cleaved caspase-3-positive photoreceptor cells were present, GFAP expression was reduced, TRPM1 expression was elevated and photoreceptor segment fragments recovered (Fig. 2c). These observations suggest that in addition to directly damaging photoreceptor cells, AUY922 treatment impairs the maintenance and renewal of photoreceptor segment fragments by suppressing the performance of RPE cells.

### AUY922 induces heat shock responses, ROS production, cell apoptosis and TRPM1 attenuation

**Fig. 3:**
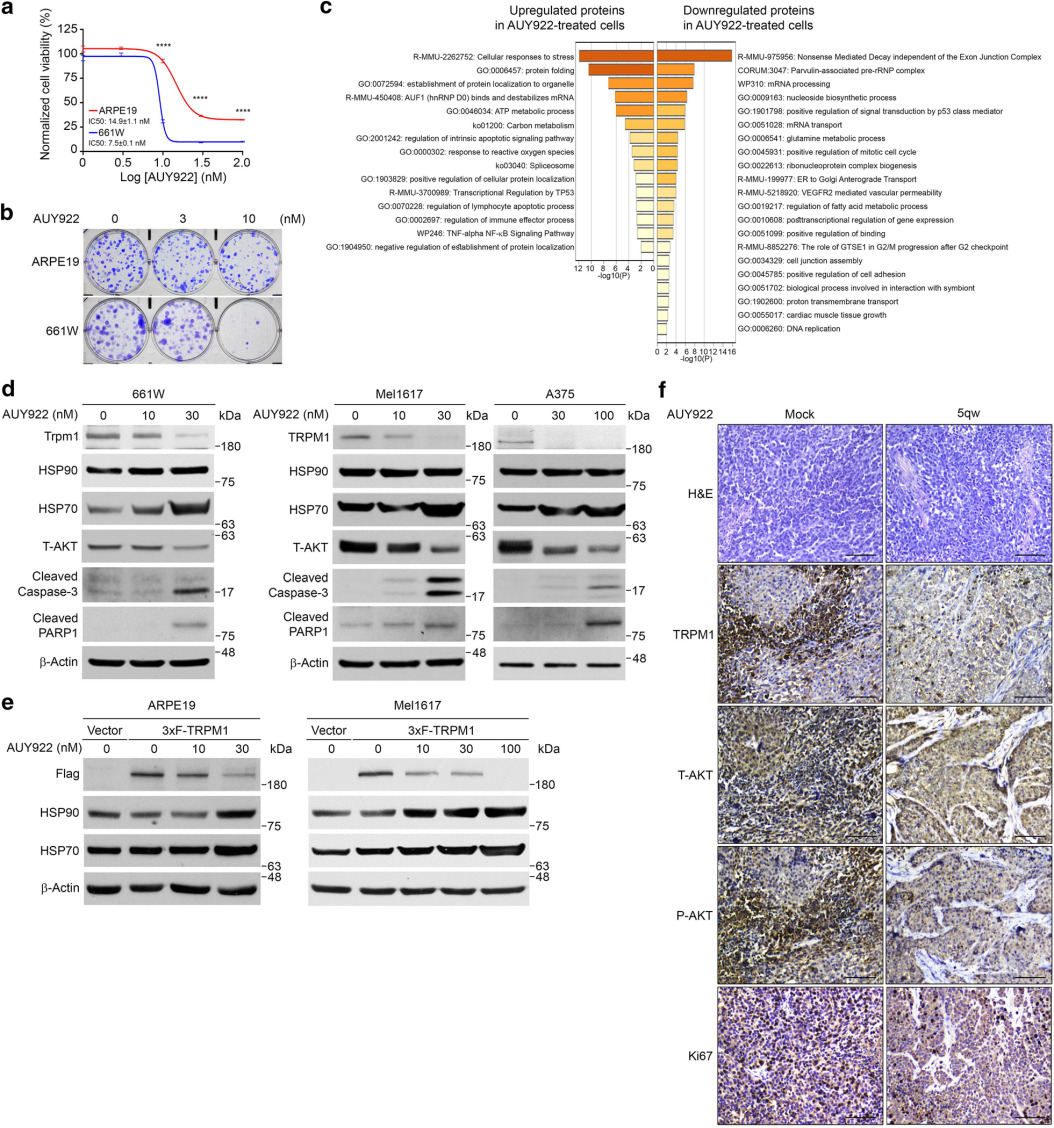
Treatment with AUY922 induced heat shock responses, cell apoptosis, ROS production and TRPM1 attenuation in vitro**. a** Viability of 661 W (blue line) and ARPE19 (red line) cells, after treatment with varying concentrations of AUY922 for 3 d. Data are presented as the mean ± s.e.m. The P values were determined by two-tailed Student’s t-test, **** P < 0.0001. *n* = 3. **b** Representative images of clonogenic growth assays for ARPE19 (top) and 661 W (bottom) cells treated with indicated concentrations of AUY922 for 10 d. Five hundred cells/well were seeded in 6-well plates. *n* = 3. **c** Functional annotation of differentially expressed proteins in AUY922-treated 661 W cells. **d** Representative western blot results of 661 W, Mel1617 and A375 cells treated with varying concentrations of AUY922 for 48 h. β-Actin was used as a loading control. *n* = 3. **e** Representative western blot results of ARPE19 (left) and Mel1617 (right) cells stably expressing 3xF-TRPM1 after treatment with varying concentrations of AUY922 for 24 h. Cells expressing an empty vector were served as controls. *n* = 3. **f** Representative images of H&E stain and IHC analysis for TRPM1, AKT (T-AKT), phospho-AKT (P-AKT) and Ki67 expression in A375 xenograft tumor samples from the AUY922 treatment experiment in Fig. 1b. Scale bar: 25 μm. *n* = 3

To explore the mechanisms of how TRPM1 mediates AUY922-induced retinal toxicity, we first examined the effect of AUY922 on cell viability and colony formation ability in ARPE19 human RPE cells and 661 W mouse cone photoreceptor cells. In tetrazolium dye MTS and clonogenic growth assays, 661 W cells exhibited higher sensitivity to AUY922 than ARPE19 cells (Fig. 3a and b), consistent with our observations that photoreceptor cells are sensitive to AUY922. Furthermore, AUY922 also suppressed the growth of A375 and Mel1617 cells, which are human melanoma cells expressing TRPM1 (Additional file 3: Figure S3a and b). To identify key proteins and cellular pathways that are differentially regulated in response to AUY922, we performed isobaric tags for relative and absolute quantitation (iTRAQ)‑based proteomic profiling on 661 W cells treated with DMSO or AUY922 and identified a number of differentially regulated molecules and pathways (Additional file 3: Figure S3c). The cell lysates collected from 661 W cells treated for 24 h with DMSO or 30 nM AUY922 were subjected to trypsin digestion. Peptides derived from DMSO- or AUY922-treated samples were labeled with iTRAQ labels 114 or 116, respectively, and subjected to LC/MS/MS analysis and protein identification. A total of 952 and 823 proteins were identified from DMSO- or AUY922-treated 661 W cells, respectively (Additional file 8: Table S1). Compared with DMSO treatment, 63 proteins were upregulated (fold change ≥ 1.2) in the AUY922-treated sample, including heat shock proteins and proteasome complexes. Sixty-two proteins with a fold change ≤ 0.833 were classified as downregulated proteins, several of which appear to be associated with the integrity of the retina, including Cav1 [43, 44], Sec13 [45, 46], Ctnna1 [47] and Tuba1a [48] (Additional file 9: Table S2). Functional annotation of differentially expressed proteins revealed that those related to stress responses, protein folding processes, regulation of apoptosis, cell cycle and growth, reactive oxygen species (ROS) response, cell junction assembly and adhesion regulation, and proton transmembrane transport were significantly enriched in AUY922-treated cells (Fig. 3c, Additional files 10, 11: Tables S3 and S4). Western blotting analysis demonstrated that AUY922 induced HSP90 and HSP70 protein expression and upregulated the levels of cleaved caspase-3 and cleaved PARP1 in 661 W, Mel1617 and A375 cells (Fig. 3d and Additional file 3: Figure S3d). ROS production also increased in 661 W cells upon AUY922 treatment (Additional file 3: Figure S3e). These results are consistent with our observation that AUY922 suppressed cell growth and validate our proteomic profiling.

TRPM1 was not identified from the proteomic analysis, which could be due to its low abundance. To test whether AUY922 downregulates the expression of TRPM1, the cells were treated with various concentrations of AUY922 for 48 h, cell lysates were collected and subjected to western blotting analysis. We found that the expression of endogenous TRPM1 and AKT (a well-known HSP90 client protein) was decreased in response to AUY922 in a dose-dependent manner in 661 W, Mel1617, and A375 cells (Fig. 3d and Additional file 3: Figure S3d). Moreover, ectopically expressed 3xFLAG-tagged TRPM1 (3xF-TRPM1 hereafter) was also downregulated after 24 h treatment with AUY922 in a dose-dependent manner in ARPE19 and Mel1617 cells (Fig. 3e and Additional file 3: Figure S3f). The levels of 3xF-TRPM1 were downregulated as early as 6 h in Mel1617 cells and 4 h in ARPE19 cells after 30 nM AUY922 was added (Additional file 3: Figure S3g). To examine whether AUY922 attenuates TRPM1 through the proteasome degradation pathway, MG132 was used to inhibit the proteasome pathway. We found that MG132 partially restored the levels of TRPM1 and AKT in AUY922-treated cells (Additional file 3: Figure S3h), supporting that AUY922 reduces TRPM1 protein via the proteasome degradation pathway. Additionally, when A375 xenograft tumors were subjected to IHC analysis, we found that TRPM1 was heterogeneous expressed in the tumors and that strong phospho-AKT expression mostly overlapped with the areas expressing TRPM1. Administration of AUY922 markedly reduced the expression of TRPM1, AKT and phospho-AKT, and eliminated actively proliferating tumor cells (Fig. 3f). These data support that AUY922 could attenuate TRPM1 and suppress cell growth by triggering cell apoptosis.

### TRPM1 is associated with the HSP90 chaperone complex

**Fig. 4:**
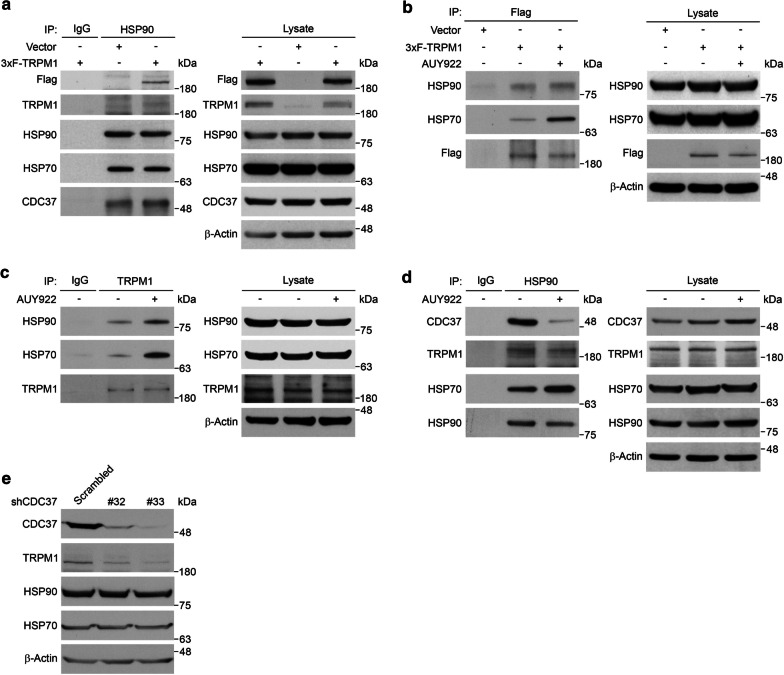
TRPM1 associated with the HSP90 chaperone complex. **a** Representative western blot results of 3xF-TRPM1, TRPM1, HSP70 and CDC37 co-immunoprecipitated (co-IP) with HSP90. Cell lysates from Mel1617 cells stably expressing either an empty vector or 3xF-TRPM1 were immunoprecipitated (IP) with anti-HSP90 conjugated agarose. Mouse IgG antibodies were served as an IP control. *n* = 3. **b** Representative western blot results of HSP90 and HSP70 co-IP with 3xF-TRPM1. Mel1617 cells stably expressing 3xF-TRPM1 were mock-treated (−) or treated with 30 nM AUY922 for 6 h (+), cell lysates were prepared and IP with anti-FLAG M2 affinity gel. Cells expressing an empty vector were served as a control. *n* = 3. **c** Representative western blot results of HSP90 and HSP70 co-IP with TRPM1. Mel1617 cells were mock-treated (−) or treated with 30 nM AUY922 for 6 h (+), cell lysates were prepared and IP with anti-TRPM1 antibodies. Mouse IgG antibodies were served as an IP control. *n* = 3. **d** Representative western blot results of CDC37, TRPM1 and HSP70 co-IP with HSP90. Mel1617 cells were mock-treated or treated with 30 nM AUY922 for 6 h (+), cell lysates were prepared and IP with anti-HSP90 conjugated agarose. Mouse IgG antibodies were served as an IP control. *n* = 3. **e** Representative western blot results of Mel1617 cells expressing either a scrambled shRNA or shRNAs specific for *CDC37*. *β*-Actin was used as a loading control. *n* = 3

AUY922 is proposed to be a highly potent ATP-competitive HSP90 inhibitor that induces the heat shock response and blocks the HSP90 chaperone. To investigate whether AUY922 attenuated TRPM1 expression through HSP90 inhibition, we first tested whether TRPM1 and heat shock proteins physically interacted. Mel1617 cells stably expressing 3xF-TRPM1 or empty vector were subjected to immunoprecipitation with anti-FLAG M2 affinity agarose gel. When analyzed by western blotting, we found that HSP90 and HSP70 associated with the anti-FLAG immunoprecipitation complex from cells expressing 3xF-TRPM1 but not from the empty vector (Additional file 4: Figure S4a). Conversely, when the cell lysates were immunoprecipitated with anti-HSP90 agarose, 3xF-TRPM1 was co-immunoprecipitated in the cells expressing 3xF-TRPM1 but not in those with the empty vector or when immunoprecipitated with the control IgG. We further confirmed that endogenous TRPM1, HSP70, and CDC37 associated with the anti-HSP90 immunoprecipitant complex but not with the control IgG precipitates (Fig. 4a).

Next, we addressed whether treatment with AUY922 interferes with the formation of TRPM1-HSP complexes. To avoid the complications resulting from TRPM1 attenuation after 24 h treatment with AUY922, we performed experiments on cells treated with 30 nM AUY922 for 6 h. Mel1617 cells expressing 3xF-TRPM1 were treated with AUY922 and cell lysates were subjected to immunoprecipitation with anti-FLAG M2 affinity agarose gel. We found that treatment with AUY922 induced heat shock responses and enhanced the association of 3xF-TRPM1 with HSP90 and HSP70 (Fig. 4b and Additional file 4: Figure S4b). Similarly, when endogenous TRPM1 was immunoprecipitated using anti-TRPM1 antibody, treatment with AUY922 enhanced the interaction of TRPM1 with HSP90 and HSP70 (Fig. 4c and Additional file 4: Figure S4c).

CDC37, a co-chaperone involved in the regulation of the HSP90 chaperone cycle, plays a role in the maturation of HSP90 clients [4, 49]. To examine the possibility that CDC37 is involved in the maturation of TRPM1, the cell lysates from Mel1617 cells that had been treated with or without 30 nM AUY922 for 6 h were immunoprecipitated with anti-HSP90 agarose. We found that treatment with AUY922 slightly increased the abundance of CDC37 in the lysates but markedly disrupted the interaction between CDC37 and HSP90 (Fig. 4d and Additional file 4: Figure S4d). Similar results were observed in ARPE19 expressing 3xF-TRPM1 cells treated with or without 30 nM AUY922 for 4 h (Additional file 4: Figure S4e). To confirm that CDC37 is required for TRPM1 maturation, we expressed shRNAs specifically against *CDC37* in Mel1617 cells. Compared to the cells expressing scrambled shRNA, the knockdown of CDC37 reduced the protein levels of TRPM1 but not the levels of HSP90 or HSP70 (Fig. 4e and Additional file 4: Figure S4f). These data suggest that TRPM1 is a bona fide HSP90 client and that AUY922 could impair the HSP90 chaperone function by disrupting the interaction of CDC37 and the HSP90 complex, which results in reduction of TRPM1.

### TRPM1 is involved in the regulation of cytosolic Ca^2+^ concentration and photoreceptor function

**Fig. 5:**
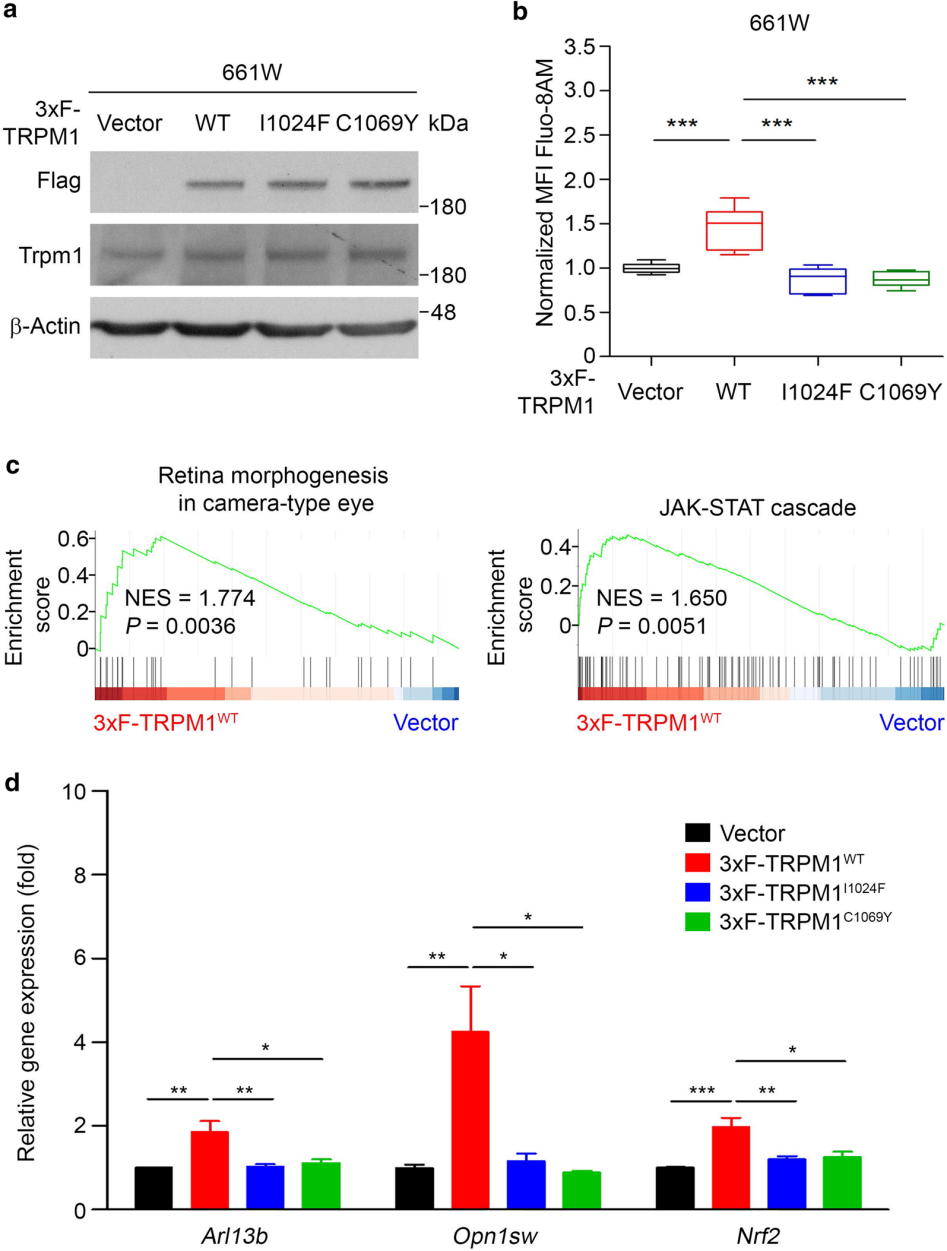
TRPM1 was involved in the regulation of cytosolic Ca^2+^ concentration and photoreceptor function. **a** Representative western blot results of cells stably expressing either an empty vector or a vector encoding 3xFLAG-tagged wild-type TRPM1 (WT), TRPM1Ile1024Phe (I1024F) or TRPM1Cys1069Tyr (C1069Y). β-Actin was used as a loading control. *n* = 3. **b** Cytosolic Ca^2+^ levels were determined by flow cytometry using the median fluorescence intensity (MFI) of the Ca^2+^ dye Fluo-8 AM in cells expressing either an empty vector or variants of TRPM1. Data are presented as the mean ± s.e.m. The P values were determined by two-tailed Student’s t-test, * P < 0.05; ** P < 0.01; *** P < 0.001. *n* = 3. **c** GSEA of the pathways enriched in TRPM1-overexpressing 661 W cells compared with control cells based on the RNA sequencing data. NES, normalized enrichment score. **d** mRNA levels of *Arl13b*, *Opn1sw* and *Nrf2* were determined in cells expressing either an empty vector or variants of TRPM1 by RT-qPCR. mRNA levels were calculated relative to those in cells expressing an empty vector. Levels of the housekeeping gene *β*-*Actin* were used as a reference. Data are presented as the mean ± s.e.m. The P values were determined by two-tailed Student’s t-test, * P < 0.05; ** P < 0.01; *** P < 0.001. *n* = 3

TRPM1 is a membrane protein with six transmembrane domains and a hydrophobic Ca^2+^-permeable pore loop between transmembrane domains 5 and 6. This constitutively open Ca^2+^ entry channel has been suggested to be switched off by the mGluR6/Go cascade in response to light stimulation [50]. Truncation mutations of the TRPM1 channel have been identified and associated with CSNB syndrome [30, 31]. However, whether other missense mutations lead to loss of function of the TRPM1 channel has not yet been proven. Therefore, we focused on two missense mutations (c.3070A > T, p. I1024F and c.3206G > A, p. C1069Y) identified in patients with CSNB, which are located at the pore loop of the TRPM1 channel [31, 51] (Additional file 5: Figure S5a). To determine the consequence of the pore loop mutations on the function of TRPM1 in vitro, we stably expressed 3xF-TRPM1 constructs containing the wild-type, I1024F and C1069Y sequences into 661 W cells (Fig. 5a and Additional file 5: Figure S5b) and quantified cytosolic Ca^2+^ levels with Fluo-8 AM. We found that the expression of wild-type TRPM1, but not the I1024F or C1069Y mutants, in 661 W cells increased the cytosolic Ca^2+^ levels compared to the vector control (Fig. 5b). Similar effects were also observed in Mel1617 cells (Additional file 5: Figure S5c and d). This indicates that TRPM1^I1024F^ and TRPM1^C1069Y^ have lost Ca^2+^ permeability. The expression of TRPM1^wildtype^, TRPM1^I1024F^ and TRPM1^C1069Y^ did not markedly alter the number or size of colonies, compared to the vector control (Additional file 5: Figure S5e).

To study the function of TRPM1 in photoreceptor cells, RNA sequencing was performed using 661 W cells stably expressing empty vector or 3xF-TRPM1. Gene set enrichment analysis (GSEA) was performed to identify affected pathways. We found that the genes associated with retinal morphogenesis in camera-type eyes and the JAK-STAT cascade pathways were enriched in TRPM1-expressing 661 W cells (Fig. 5c, and Additional file 12: Table S5). The genes that regulate retinal morphogenesis and the JAK-STAT cascade are shown in the heatmap and hierarchical clustering analysis (Additional file 5: Figure S5f) and validated by quantitative reverse transcription PCR (RT-qPCR) with specific primer pairs for *Hes1* and *Lrp5* (Additional file 5: Figure S5g). Based on the gene signature, we further investigated whether the overexpression of TRPM1 had any effects on the expression of genes linked to ciliopathies and photo-oxidative stress responses of photoreceptor cells, as well as whether the Ca^2+^ permeability of TRPM1 was required. mRNAs collected from 661 W cells carrying control vector, TRPM1^wildtype^, TRPM1^I1024F^, or TRPM1^C1069Y^ were subjected to RT-qPCR analysis for quantify the expression of *Arl13b* (encodes ADP-ribosylation factor-like GTPase 13B for retinogenesis and segmentation of photoreceptor cells), *Opn1sw* (encodes short-wave-sensitive opsin 1) and *Nrf2* (encodes Nrf2 for reducing ROS) as well as photoreceptor cilia markers, including *Cep164* (distal centriolar appendage marker), *Rpgrip1l* (transition zone marker) and *Ift88* (axonemal marker) [52–55]. The data showed that TRPM1^wildtype^, but not TRPM1^I1024F^ or TRPM1^C1069Y^, upregulated the expression of *Opn1sw*, *Arl13b*, and *Nrf2* (Fig. 5d). Expression of wild-type or mutants TRPM1 did not alter the expression of *Cep164*, *Rpgrip1l*, or *Ift88* in 661 W cells (Additional file 5: Figure S5h). This indicates that TRPM1 could play a function in photoreceptor cells and might be involved in conferring light-induced ROS stress. Additionally, these data further support that TRPM1^I1024F^ and TRPM1^C1069Y^ are functionally defective mutations.

### TRPM1 mediates AUY922-induced apoptosis, ROS production and cell growth suppression

**Fig. 6:**
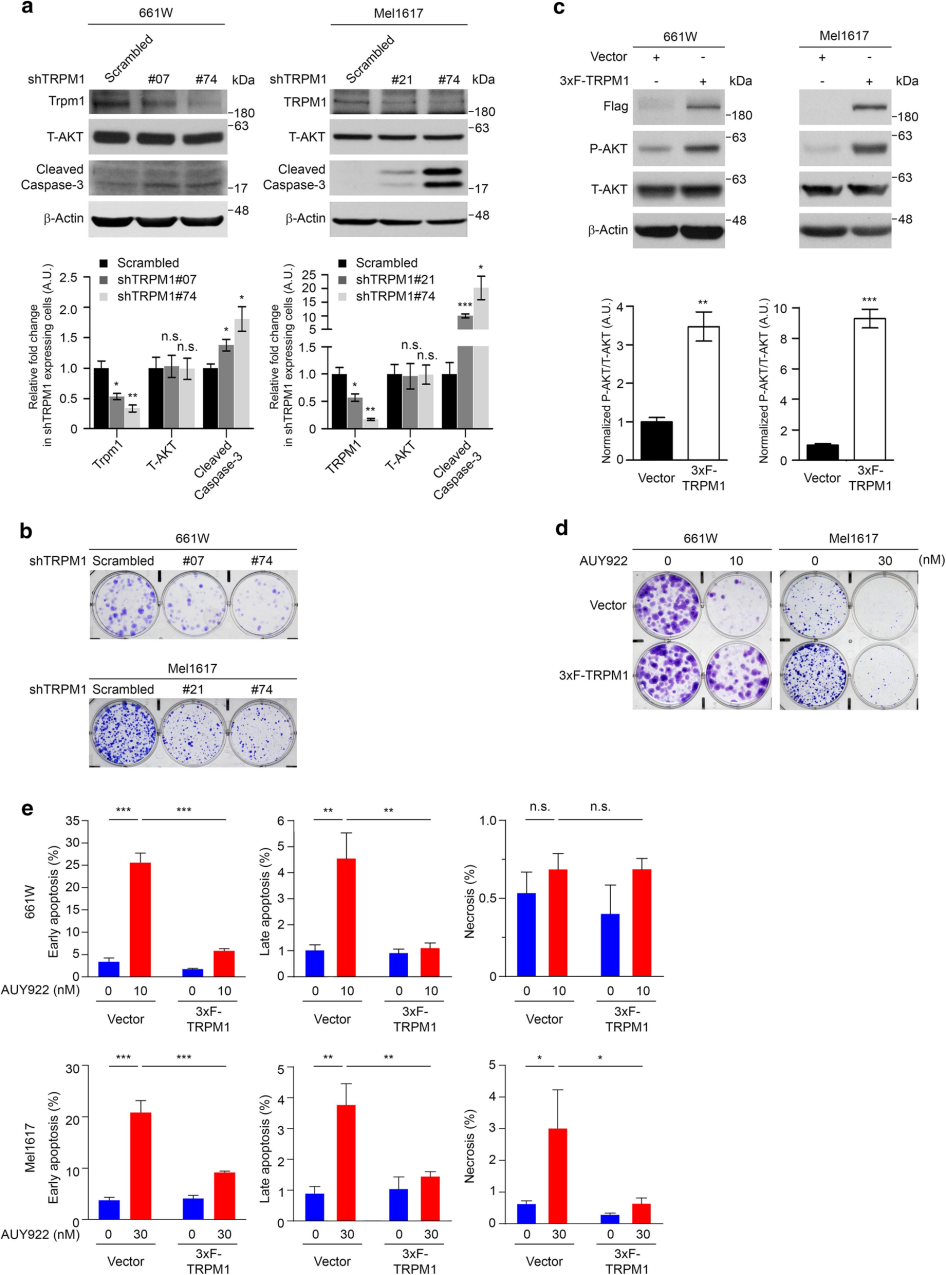
TRPM1 mediated AUY922-induced apoptosis, ROS production and cell growth suppression. **a** Representative western blot results of 661 W (left) and Mel1617 (right) cells stably expressing either a scrambled shRNA or shRNAs specific for *TRPM1*. *β*-Actin was used as a loading control. Quantification was performed for three independent experiments. Data are presented as the mean ± s.e.m. The P values were determined by two-tailed Student’s t-test, * P < 0.05; ** P < 0.01; *** P < 0.001; n.s. = not significant. *n* = 3. **b** Representative images of clonogenic growth assays in *TRPM1*-knockdown cells described in Fig. 6a. Fifteen hundred 661 W cells/well and 3000 Mel1617 cells/well were seeded in 6-well plates for 10 d. *n* = 3. **c** Representative western blot results of cells stably expressing either an empty vector or 3xF-TRPM1. *β*-Actin was used as a loading control. *n* = 3. Quantification analysis was performed for three independent experiments. Data are presented as the mean ± s.e.m. Significances were determined by two tailed Student’s t-test, n.s. = not significant. **d** Representative images of clonogenic growth assays in cells stably expressing an empty vector or 3xF-TRPM1, after treatment with the indicated concentrations of AUY922 for 10 d. Five hundred 661 W cells/well and 2000 Mel1617 cells/well were seeded in 6-well plates. *n* = 3. **e** Apoptosis analysis was performed in cells stably expressing an empty vector or 3xF-TRPM1 after treatment with the indicated concentrations of AUY922 for 24 h. Data are presented as the mean ± s.e.m. The P values were determined by two-tailed Student’s t-test, *** P < 0.001. *n* = 3

Given that AUY922 inhibits cell growth and attenuates TRPM1 expression in vivo and in vitro, it is expected that TRPM1 plays a role in mediating AUY922-induced cell apoptosis and growth suppression. To address the role of TRPM1 in cell growth, we used TRPM1-specific shRNA#74 to downregulate TRPM1 in both human and mouse cell lines, and additional shRNA#07 for mouse Trpm1 and shRNA#21 for human TRPM1 to exclude potential off-target effects. 661 W cells expressing TRPM1 shRNA#07 or shRNA#74 were subjected to western blot and clonogenic growth assays. We found that knockdown of TRPM1 elevated the levels of cleaved caspase-3 and inhibited colony formation, compared to cells expressing a scrambled shRNA (Fig. 6a and b). Similar results were observed in Mel1617, ARPE19, and A375 cells expressing human TRPM1-specific shRNAs (Fig. 6a and b, Additional file 6: Figure S6a and b). Next, the effects of ectopically expressed TRPM1 on cell growth and AUY922 sensitivity were tested. We found that the expression of 3xF-TRPM1 upregulated phospho-AKT levels in 661 W and Mel1617 cells (Fig. 6c) but not discernably in ARPE19 cells (Additional file 6: Figure S6c). In addition, TRPM1 alleviated the inhibitory effect of AUY922 on colony formation in 661 W and Mel1617 cells (Fig. 6d) but only marginally in ARPE19 cells (Additional file 6: Figure S6d). TRPM1 also reduced the percentages of apoptotic (early and late) cells and ROS production upon AUY922 treatment in 661 W cells (Fig. 6e, Additional file 6: Figure S6e and f). Similar results were observed in Mel1617 cells, and the expression of 3xF- TRPM1 decreased the percentages of both apoptotic (late and early) and necrotic cells induced by AUY922 (Fig. 6e and Additional file 6: Figure S6e). These data suggest that TRPM1 is critical for cell growth and mediates AUY922-induced apoptosis and growth suppression.

## Discussion

Clinically, abnormal electroretinograms reveal that vision impairment induced by AUY922 mainly occurs due to retinal dysfunction. In vivo models show that AUY922 induces retinal injury and photoreceptor cell apoptosis, dysregulates photoreceptor and RPE cells, and attenuates the expression of TRPM1, which is critical for the neurotransmission pathway. In vitro models show that AUY922 induces heat shock responses, apoptosis, ROS production, cell growth suppression and TRPM1 attenuation. Mechanistically, we determined that TRPM1 interacts with heat shock proteins and is an HSP90 client. AUY922 attenuates TRPM1 expression by disrupting the CDC37-HSP90 chaperone complex. Functionally, TRPM1 is involved in cytosolic Ca^2+^ concentration regulation, photoreceptor morphogenesis and function, and ROS elimination pathways (Additional file 7: Figure S7). Our study presents evidence that AUY922 targets TRPM1 to induce retinal toxicity.

The molecular pathology associated with AUY922-induced ocular toxicity has not been well characterized. The biological consequence of AUY922, a potent anti-cancer drug, on suppressing cell growth is well documented in vitro and in vivo. Clinically, AUY922 as a single regimen or in combination with other compounds has been evaluated in many types of human cancers and reported to induce ocular AEs [16–19, 56–60]. AUY922-induced cell apoptosis has been reported in retinas from albino Sprague Dawley rats intravenously treated with 10.0 mg/kg/3qw for 2 weeks [21]. In our mouse models, upon AUY922 treatment, retinal sections showed upregulation of cell apoptosis and GFAP, downregulation of TRPM1 and retinoid isomerohydrolase, and dysregulation of the outer segment of photoreceptors. Ten days after drug withdrawal, these alterations were reversed. This suggests that these markers represent the effects of AUY922 on the retina and can be used to evaluate the retinal toxicity of drugs preclinically.

The IS/OS layer consists of the outer segment (OS), connecting cilium (CC) and inner segment (IS) of photoreceptors. The OS where photoreceptors collect and convert light into electrical signals facilitates phototransduction. The IS that connects to the OS by CC is responsible for ATP production. The renewal and shedding of membranous discs in the outer segments occur daily to ensure maximum photosensitivity of photoreceptors and maintain the health of the outer retina. The new discs are synthesized at the proximal end and transported to the distal end of the OS, whereas the old discs are shed at the distal end of the OS and phagocytosed by the RPE [40, 61, 62]. These discs are completely replaced once every 10 d in mice [63]. Our data support that AUY922 disrupted the renewal and shedding processes mediated by RPE, which dysregulated the outer segments and impaired phototransduction. Furthermore, the terminus of the photoreceptor projects into the synaptic region, which consists of synaptic vesicles and a ribbon synapse for synaptic transmission from photoreceptors to bipolar cells and other secondary neurons and forms OPLs [64]. We found that, in OPL, AUY922 treatment reduced the expression of TRPM1, which is critical for synapse formation and neurotransmission pathways. This indicates that AUY922 is detrimental to both phototransduction and neurotransmission pathways and impairs vision.

Several elegant studies have revealed that the intrinsic ATPase activity of the N-terminal domain triggers the conformational switch of HSP90, which is necessary for the folding and activation of client proteins in eukaryotic cells [65]. The binding of unfolded clients with and the release of mature clients from HSP90 are regulated by other co-chaperones, such as HSP70 and CDC37. HSP70 has high binding affinity for unfolded clients and brings unfolded clients into the HSP90 complex [3]. CDC37 has been suggested to function in conformational maturation, protein folding, and acquisition of functional states for a large variety of clients, including protein kinases and non-kinase receptors [66, 67]. Several small molecule ATP-mimetics, such as 17-AAG and AUY922, have been developed to inhibit the HSP90 complex and proposed as anti-cancer agents [68–71]. We found that AUY922 instantly induces heat shock responses, including upregulation of HSP expression and promotion of HSP-TRPM1 interactions. After 24 h in the presence of AUY922, the expression of TRPM1 decreased. Previous studies have suggested that HSP90 inhibitor-induced heat shock responses prevent or reverse client protein misfolding and ultimately direct misfolded clients to polyubiquitylation and proteasomal degradation [72–74]. Additionally, we present several lines of evidence indicating that TRPM1 is a bona fide HSP90 client. First, AUY922 attenuates the expression of TRPM1 in vitro and in vivo. Second, TRPM1 physically associates with the HSP complex. Third, CDC37 is a TRPM1 co-chaperone and is required for stabilizing TRPM1 expression. In addition to the inhibitory effect of AUY922 on the ATPase activity of HSP90, our data further show that AUY922 could disrupt the interaction between CDC37 and the HSP90 complex to impair chaperone function and result in TRPM1 loss. The effect of AUY922 on the interaction of HSP90 with other co-chaperones, such as HOP and AHA1, will be investigated in the future.

TRPM1 is a constitutively open Ca^2+^ entry channel on the plasma membrane with six transmembrane domains. The function of TRPM1 in mediating visual performance has been associated with its Ca^2+^ entry channel function. Ca^2+^ is a critical second messenger involved in a variety of physiological processes, including neuronal transmission, cell motility, and cell growth. In this report, we have validated that TRPM1 functions in cytosolic Ca^2+^ concentration regulation, photoreceptor morphogenesis, and ROS elimination pathways. Mutations at the pore loop of TRPM1 block the Ca^2+^ entry and attenuate the expression of *Opn1sw*, *Arl13b*, and *Nrf2*, supporting that TRPM1^I1024F^ and TRPM1^C1069Y^ are functionally defective mutants and could be pathogenic for CSNB.

We identified pathological characteristics that are strongly associated with AUY922-induced retinal toxicity, identified TRPM1 as a novel HSP90 client, and uncovered the role of TRPM1 in mediating AUY922-induced apoptosis and cell growth suppression, and in the development and integrity of photoreceptor cells. Our study could also provide a molecular-based platform and guideline for evaluating drug-induced retinal toxicity preclinically.

## Conclusions

Our study demonstrates the pathology of AUY922-induced retinal toxicity in vivo. TRPM1 is an HSP90 client and regulates photoreceptor morphology and function, mediating AUY922-induced cytotoxicity.

## Supplementary Information

**Additional file 1: Figure S1. a** Full-field electroretinogram (ffERG) of a patient with refractory gastrointestinal stromal tumor without AUY922 treatment. OD: right eye, OS: left eye. **b** Quantification of tumor volume in nude mice bearing xenograft tumors of A375 cells treated with mock (*n* = 6), 25 mg/kg AUY922 three times weekly (3qw, *n* = 6), or five times weekly (5qw, *n* = 6) for 2 w by intraperitoneal injection. Data are presented as the mean ± s.e.m. The P values were determined by two-tailed Student’s t-test, *** P < 0.001.**c** Body weight of the tumor-bearing mice in Figure S1b. Data are presented as the mean ± s.e.m. **d** Representative images of H&E staining of mouse eye samples from the AUY922 treatment experiment in Fig. 1b. Scale bar: 25 μm. *n* = 3. **e** Quantification of INL (left) and ONL (right) thickness of Figure S1d. The thickness of the INL and ONL was measured in H&E-stained sections at comparable locations. Data are presented as the mean ± s.e.m. Significances were determined by two-tailed Student’s t-test, ns: not significant. *n* = 3. **f** Non-cropped images of Fig. 1b. Boxed area was magnified, leveled, presented in Fig. 1b. bar: 25 μm.

**Additional file 2: Figure S2.** Representative images of H&E staining and TUNEL assays for REP flat mounts from the AUY922 treatment experiment in Fig. 1b. Scale bar: 25 μm. *n* = 3.

**Additional file 3: Figure S3. a** Viability of A375 (blue line) and Mel1617 (red line) cells, after treatment with varying concentrations of AUY922 for 3 d. Data are presented as the mean ± s.e.m. (*n* = 3). **b** Representative images of clonogenic growth assays in Mel1617 (top) and A375 (bottom) cells treated with the indicated concentrations of AUY922 for 10 d. Three hundred cells/well were seeded in 6-well plates. *n* = 3. **c** Strategy for iTRAQ proteomic profiling of DMSO- and AUY922-treated 661 W cells. **d** Quantification of the AUY922 treatment experiments in 661 W, Mel1617 and A375 cells, related to Fig. 3d. Data are presented as the mean ± s.e.m. The P values were determined by two-tailed Student’s t-test, * P < 0.05; ** P < 0.01. *n* = 3. **e** ROS production assays were performed in 661 W cells treated with AUY922 for 48 h. Cells were labeled with DCFDA (20 μM) and analyzed by flow cytometry. Data are presented as the mean ± s.e.m. The P values were determined by two-tailed Student’s t-test, ** P < 0.01; n.s. = not significant. *n* = 3. **f** Quantification of the AUY922 treatment experiments in ARPE19 and Mel1617 cells expressing 3xF-TRPM1, related to Fig. 3e. Data are presented as the mean ± s.e.m. The P values were determined by two-tailed Student’s t-test, * P < 0.05; ** P < 0.01; *** P < 0.001. *n* = 3. **g** Representative western blot results of ARPE19 and Mel1617 cells stably expressing either an empty vector or 3xF-TRPM1 after treatment with 30 nM AUY922 for different time points. Quantification was performed for three independent experiments. Data are presented as the mean ± s.e.m. The P values were determined by two-tailed Student’s t-test, * P < 0.05; ** P < 0.01; *** P < 0.001; n.s. = not significant. *n* = 3. **h** Representative western blot results of 661 W cells pretreated with 30 nM AUY922 for 48 h and treated with 100 nM MG132 for 4 h. Quantification was performed for three independent experiments. Data are presented as the mean ± s.e.m. The P values were determined by two-tailed Student’s t-test, * P < 0.05; ** P < 0.01. *n* = 3.

**Additional file 4: Figure S4. a** Representative western blot results of HSP90 and HSP70 co-IP with 3xF-TRPM1. Cell lysates were prepared from Mel1617 cells stably expressing either an empty vector or 3xF-TRPM1 and IP with anti-FLAG M2 affinity gel. *n* = 3. **b** Quantification analysis, related to Fig. 4b, was performed for three independent experiments. Data are presented as the mean ± s.e.m. The P values were determined by two-tailed Student’s t-test, * P < 0.05; ** P < 0.01. *n* = 3. **c** Quantification analysis, related to Fig. 4c, was performed for three independent experiments. Data are presented as the mean ± s.e.m. The P values were determined by two-tailed Student’s t-test, ** P < 0.01; *** P < 0.001. *n* = 3. **d** Quantification analysis, related to Fig. 4d, was performed for three independent experiments. Data are presented as the mean ± s.e.m. The P values were determined by two-tailed Student’s t-test, ** P < 0.01; n.s. = not significant. *n* = 3. **e** Representative western blot results of CDC37, 3xF-TRPM1 and HSP70 co-IP with HSP90. ARPE19 cells expressing 3xF-TRPM1 were mock-treated (-) or treated with 30 nM AUY922 for 4 h ( +). Cell lysates were prepared and IP with anti-HSP90 conjugated agarose. Mouse IgG antibodies were served as an IP control. Quantification was performed for three independent experiments. Data are presented as the mean ± s.e.m. The P values were determined by two-tailed Student’s t-test, ** P < 0.01; *** P < 0.001; n.s. = not significant. *n* = 3. **f** Quantification analysis, related to Fig. 4e, was performed for three independent experiments. Data are presented as the mean ± s.e.m. The P values were determined by two-tailed Student’s t-test, ** P < 0.01; n.s. = not significant. *n* = 3.

**Additional file 5: Figure S5. a** Schematic representation of TRPM1 protein. **b** Quantification analysis, related to Fig. 5a, was performed for three independent experiments. Data are presented as the mean ± s.e.m, *n* = 3. **c** Representative western blot results of cells stably expressing either an empty vector or a vector encoding 3xFLAG-tagged wild-type TRPM1 (WT), TRPM1Ile1024Phe (I1024F) or TRPM1Cys1069Tyr(C1069Y). β-Actin was used as a loading control. Quantification was performed for three independent experiments. Data are presented as the mean ± s.e.m. *n* = 3. **d** Cytosolic Ca^2+^ levels were determined by flow cytometry and expressed as the MFI of the Ca^2+^ dye Fluo-8 AM in cells expressing either an empty vector or variants of TRPM1. Data are presented as the mean ± s.e.m. The P values were determined by two-tailed Student’s t-test, * P < 0.05; ** P < 0.01; *** P < 0.001. n = 3. **e** Representative images of clonogenic growth assays of 661 W cells expressing an empty vector or variants of TRPM1. Five hundred cells/well were seeded in 6-well plates for 10 d. *n* = 3. **f** Heatmaps of gene related to retinal morphogenesis in camera-type eyes (left) and JAK-STAT cascade (right) comparing TRPM1 overexpressing 661 W cells with control cells. **g** mRNA levels of *Lrp5* and *Hes1* were determined in cells expressing either an empty vector or variants of TRPM1 by RT-qPCR. mRNA levels were calculated relative to those in cells expressing an empty vector. Levels of the housekeeping gene *β*-*Actin* were used as a reference. Data are presented as the mean ± s.e.m. The P values were determined by two-tailed Student’s t-test, * P < 0.05. *n* = 3. **h** mRNA levels of *lft88*, *Cep164* and *Rpgrip1l* were determined in cells expressing either an empty vector or variants of TRPM1 by RT-qPCR. mRNA levels were calculated relative to those in cells expressing an empty vector. Levels of the housekeeping gene *β*-*Actin* were used as a reference. Data are presented as the mean ± s.e.m. The P values were determined by two-tailed Student’s t-test, ns: not significant. *n* = 3.

**Additional file 6: Figure S6. a** Representative western blot results of ARPE19 (left) and A375 (right) cells expressing either scrambled shRNA or shRNAs specific for *TRPM1*. Quantification analysis was performed for three independent experiments. Data are presented as the mean ± s.e.m. The P values were determined by two-tailed Student’s t-test, * P < 0.05; ** P < 0.01; n.s. = not significant. *n* = 3. **b** Representative images of clonogenic growth assays in *TRPM1-*knockdown cells described in Figure S6a. Five hundred ARPE19 (top) and 3000 A375 (bottom) cells were seeded in 6-well plates for 10 d. *n* = 3. **c** Representative western blot results of ARPE19 cells expressing either an empty vector or 3xF-TRPM1. Quantification analysis was performed for three independent experiments. Data are presented as the mean ± s.e.m. Significances were determined by two tailed Student’s t-test, n.s. = not significant. *n* = 3. **d** Representative images of clonogenic growth assays in ARPE19 cells stably expressing an empty vector or 3xF-TRPM1 after treatment with the indicated concentrations of AUY922 for 10 d. Five hundred cells/well were seeded in 6-well plates. *n* = 3. **e** Representative flow cytometry plots for apoptosis analyses are shown. Cells stably expressing an empty vector or 3xF-TRPM1 were treated with the indicated concentrations of AUY922 for 24 h before apoptosis analyses were performed. *n* = 3. **f** ROS levels were measured in 661 W cells treated with AUY922 for 48 h. Cells were labeled with DCFDA (20 μM) and analyzed by flow cytometry. ROS levels are expressed as fold change. Data are presented as the mean ± s.e.m. The P values were determined by two-tailed Student’s t-test, ** P < 0.01. *n* = 3.

**Additional file 7: Figure S7.** A schematic representation of the mechanism of AUY922-induced retinal toxicity through attenuation of TRPM1.

**Additional file 8: Table S1.** Protein identification in 661 W cells treated with DMSO or AUY922.

**Additional file 9: Table S2.** Differentially expressed proteins in AUY922-treated cells.

**Additional file 10: Table S3.** Functional annotation of upregulated proteins in AUY922-treated cells.

**Additional file 11: Table S4.** Functional annotation of downregulated proteins in AUY922-treated cells.

**Additional file 12: Table S5.** Genes differentially expressed in 661 W cells overexpressing 3xF-TRPM1 compared to empty vectors.

## Data Availability

The data underlying this article are available in Gene Expression Omnibus database under accession number GSE165691 and in the Additional file 12: Table S5. All materials are available upon reasonable request to chshen@nhri.edu.tw.

## Abbreviations

17-AAG: 17-Allyl-17-demethoxygeldanamycin
AEs: Adverse events
Arl13b: ADP-ribosylation factor-like GTPase 13B
CDC37: Cell division cycle 37
CSNB: Congenital stationary night blindness syndrome
ERG: Electroretinogram
ffERG: Full-field electroretinogram
GCL: Ganglion cell layer
GFAP: Glial fibrillary acidic protein
GIST: Gastrointestinal stromal tumor
HSP70: Heat shock protein 70
HSP90: Heat shock protein 90
INL: Inner nuclear layer
IS/OS: The junction between the photoreceptor's inner and outer segments
mGluR6: Metabotropic glutamate receptor 6
Nrf2: NF-E2-related factor 2
ONL: Outer nuclear layer
OPL: Outer plexiform layer
Opn1sw: Short-wave-sensitive opsin 1
PNA: Rhodamine-labeled peanut agglutinin
RPE: Retinal pigment epithelia
TRPM1: Transient receptor potential cation channel subfamily M member 1
