# Supplementary figures and images for: AUY922 induces retinal toxicity through attenuating TRPM1
Source: J Biomed Sci. 2021 Jul 23;28:55. doi: 10.1186/s12929-021-00751-5 (PMC8306347; doi:10.1186/s12929-021-00751-5)

Figure S1

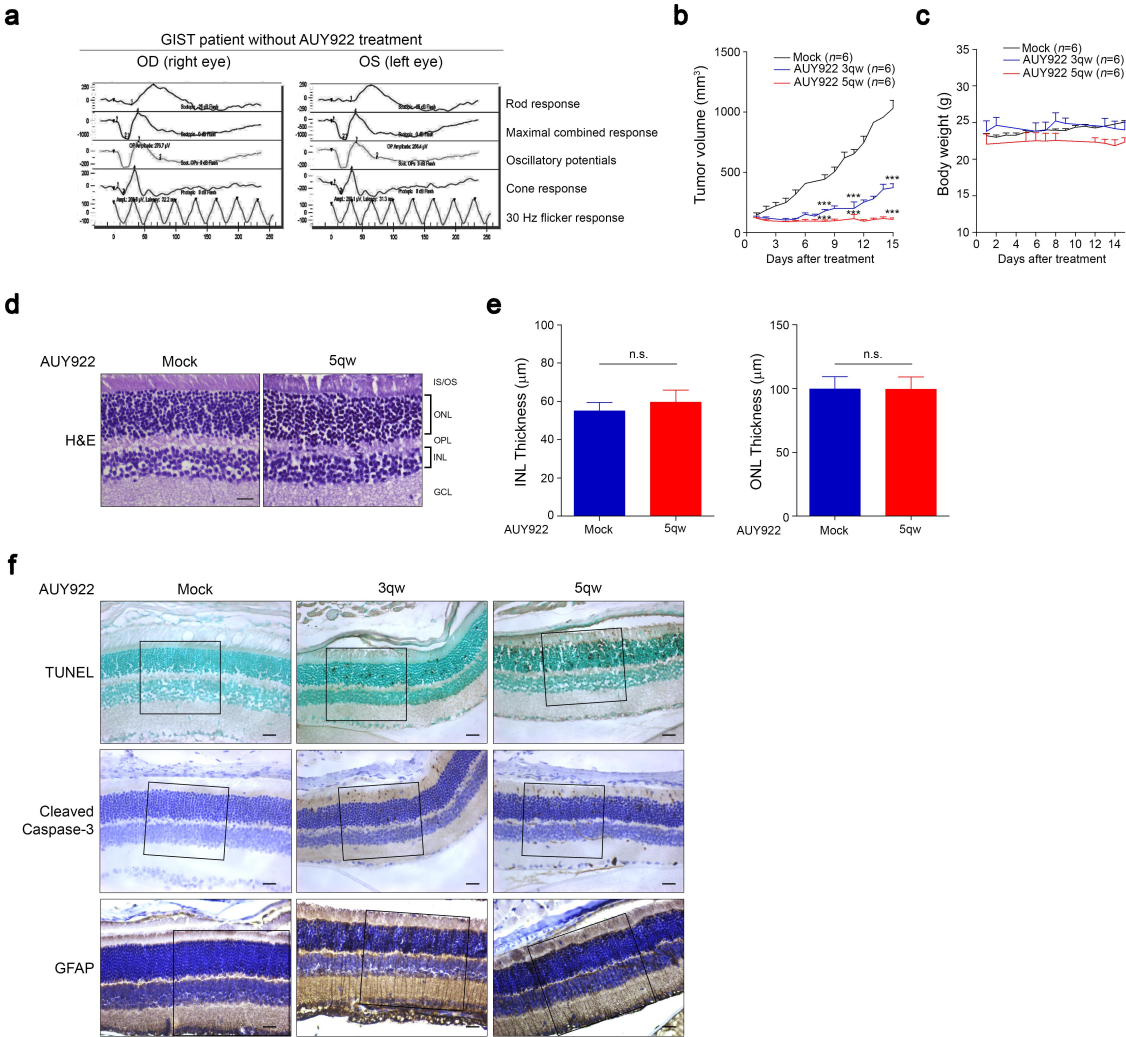

Supplement: Supplementary file 1 — Additional file 1: Figure S1. a Full-field electroretinogram (ffERG) of a patient with refractory gastrointestinal stromal tumor without AUY922 treatment. OD: right eye, OS: left eye. b Quantification of tumor volume in nude mice bearing xenograft tumors of A375 cells treated with mock (n = 6), 25 mg/kg AUY922 three times weekly (3qw, n = 6), or five times weekly (5qw, n = 6) for 2 w by intraperitoneal injection. Data are presented as the mean ± s.e.m. The P values were determined by two-tailed Student’s t-test, *** P < 0.001.c Body weight of the tumor-bearing mice in Figure S1b. Data are presented as the mean ± s.e.m. d Representative images of H&E staining of mouse eye samples from the AUY922 treatment experiment in Fig. 1b. Scale bar: 25 μm. n = 3. e Quantification of INL (left) and ONL (right) thickness of Figure S1d. The thickness of the INL and ONL was measured in H&E-stained sections at comparable locations. Data are presented as the mean ± s.e.m. Significances were determined by two-tailed Student’s t-test, ns: not significant. n = 3. f Non-cropped images of Fig. 1b. Boxed area was magnified, leveled, presented in Fig. 1b. bar: 25 μm. [file 12929_2021_751_MOESM1_ESM.pdf]

AUY922

Mock

5qw

H&E

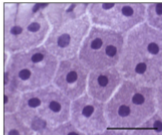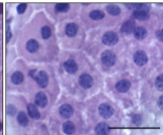

TUNEL

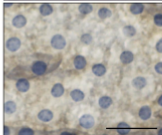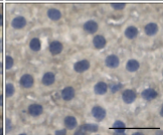

Supplement: Supplementary file 2 — Additional file 2: Figure S2. Representative images of H&E staining and TUNEL assays for REP flat mounts from the AUY922 treatment experiment in Fig. 1b. Scale bar: 25 μm. n = 3. [file 12929_2021_751_MOESM2_ESM.pdf]

**Figure S3**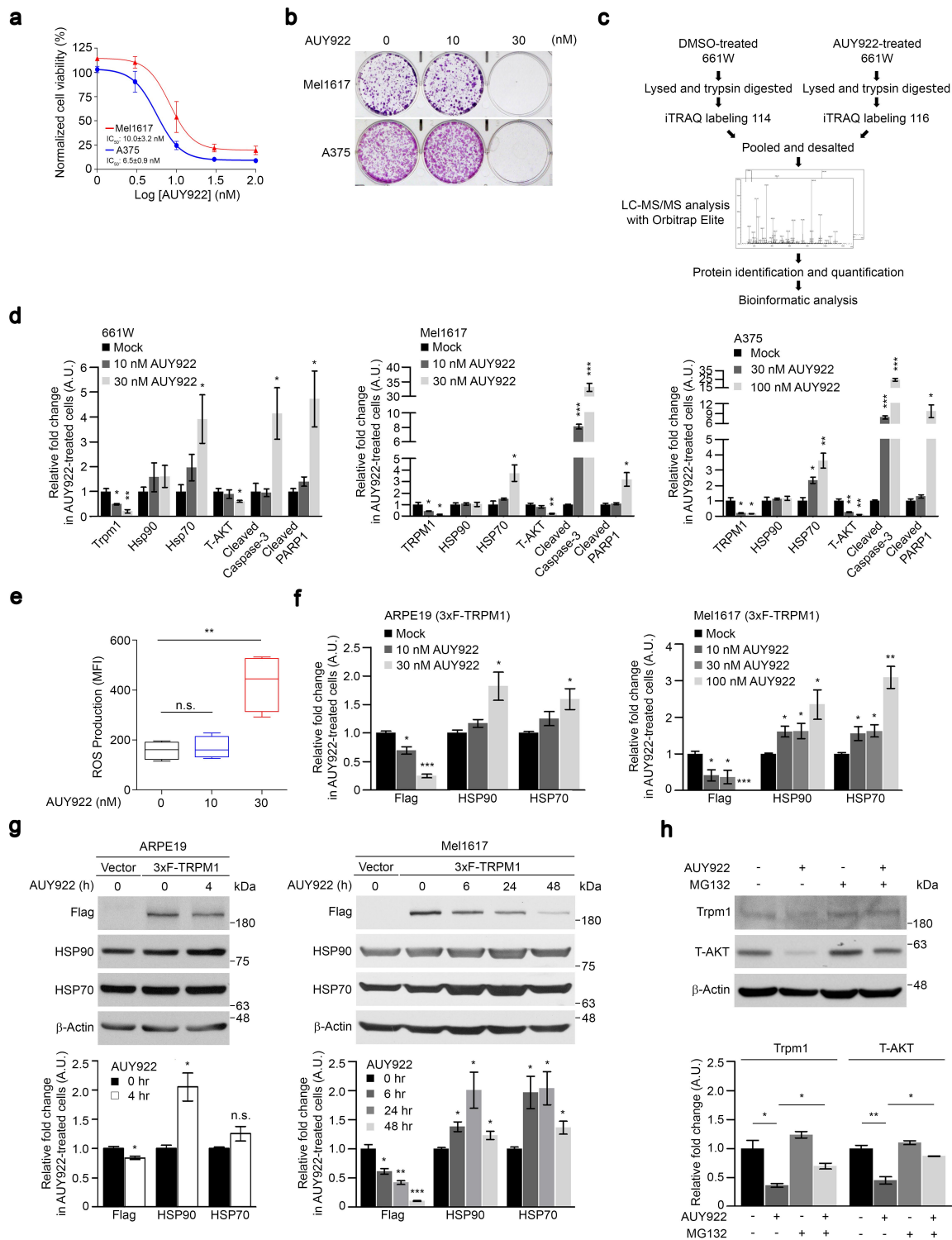

Supplement: Supplementary file 3 — Additional file 3: Figure S3. a Viability of A375 (blue line) and Mel1617 (red line) cells, after treatment with varying concentrations of AUY922 for 3 d. Data are presented as the mean ± s.e.m. (n = 3). b Representative images of clonogenic growth assays in Mel1617 (top) and A375 (bottom) cells treated with the indicated concentrations of AUY922 for 10 d. Three hundred cells/well were seeded in 6-well plates. n = 3. c Strategy for iTRAQ proteomic profiling of DMSO- and AUY922-treated 661 W cells. d Quantification of the AUY922 treatment experiments in 661 W, Mel1617 and A375 cells, related to Fig. 3d. Data are presented as the mean ± s.e.m. The P values were determined by two-tailed Student’s t-test, * P < 0.05; ** P < 0.01. n = 3. e ROS production assays were performed in 661 W cells treated with AUY922 for 48 h. Cells were labeled with DCFDA (20 μM) and analyzed by flow cytometry. Data are presented as the mean ± s.e.m. The P values were determined by two-tailed Student’s t-test, ** P < 0.01; n.s. = not significant. n = 3. f Quantification of the AUY922 treatment experiments in ARPE19 and Mel1617 cells expressing 3xF-TRPM1, related to Fig. 3e. Data are presented as the mean ± s.e.m. The P values were determined by two-tailed Student’s t-test, * P < 0.05; ** P < 0.01; *** P < 0.001. n = 3. g Representative western blot results of ARPE19 and Mel1617 cells stably expressing either an empty vector or 3xF-TRPM1 after treatment with 30 nM AUY922 for different time points. Quantification was performed for three independent experiments. Data are presented as the mean ± s.e.m. The P values were determined by two-tailed Student’s t-test, * P < 0.05; ** P < 0.01; *** P < 0.001; n.s. = not significant. n = 3. h Representative western blot results of 661 W cells pretreated with 30 nM AUY922 for 48 h and treated with 100 nM MG132 for 4 h. Quantification was performed for three independent experiments. Data are presented as the mean ± s.e.m. The P values were determined by two- [file 12929_2021_751_MOESM3_ESM.pdf]

**Figure S4**

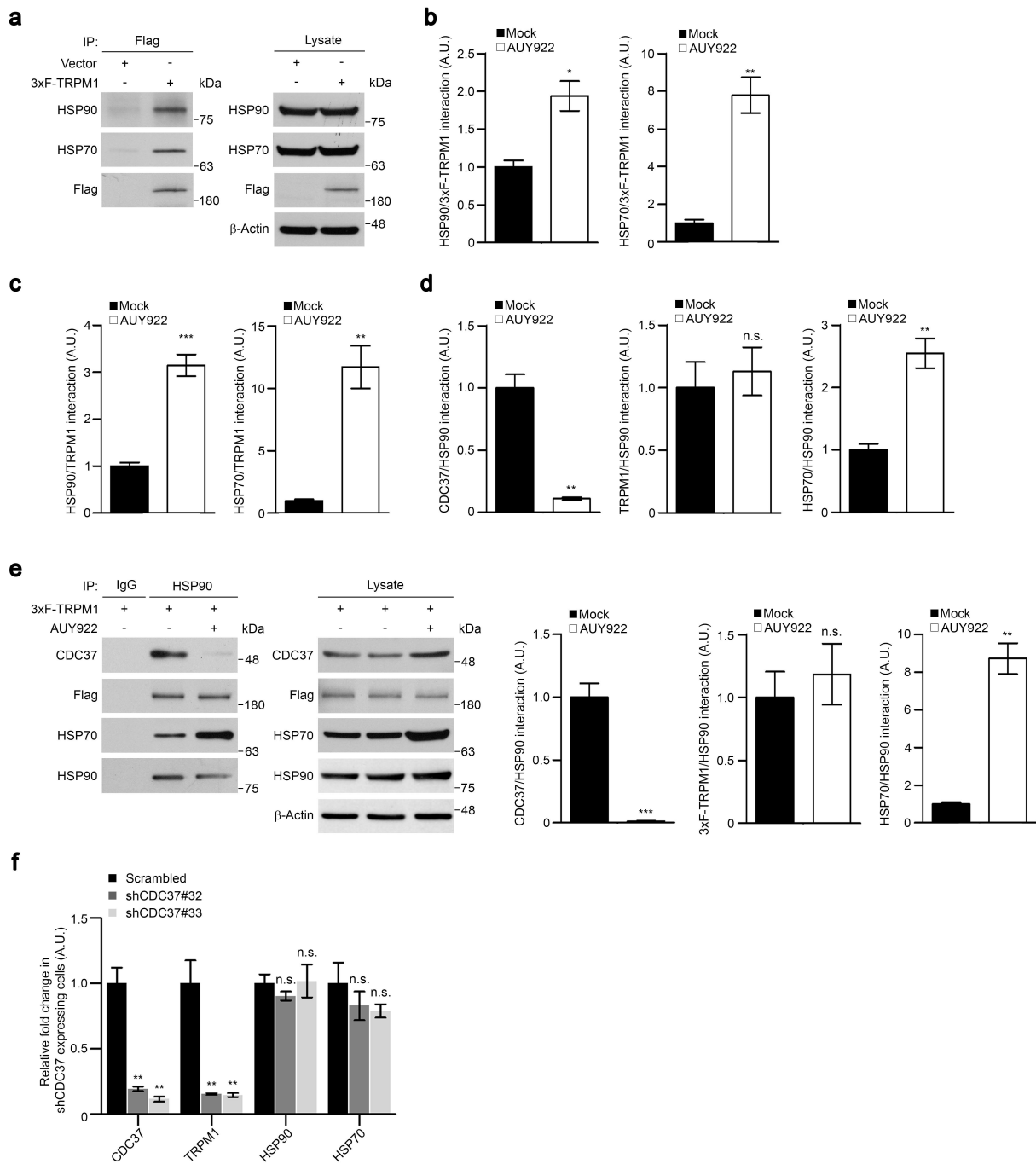

Supplement: Supplementary file 4 — Additional file 4: Figure S4. a Representative western blot results of HSP90 and HSP70 co-IP with 3xF-TRPM1. Cell lysates were prepared from Mel1617 cells stably expressing either an empty vector or 3xF-TRPM1 and IP with anti-FLAG M2 affinity gel. n = 3. b Quantification analysis, related to Fig. 4b, was performed for three independent experiments. Data are presented as the mean ± s.e.m. The P values were determined by two-tailed Student’s t-test, * P < 0.05; ** P < 0.01. n = 3. c Quantification analysis, related to Fig. 4c, was performed for three independent experiments. Data are presented as the mean ± s.e.m. The P values were determined by two-tailed Student’s t-test, ** P < 0.01; *** P < 0.001. n = 3. d Quantification analysis, related to Fig. 4d, was performed for three independent experiments. Data are presented as the mean ± s.e.m. The P values were determined by two-tailed Student’s t-test, ** P < 0.01; n.s. = not significant. n = 3. e Representative western blot results of CDC37, 3xF-TRPM1 and HSP70 co-IP with HSP90. ARPE19 cells expressing 3xF-TRPM1 were mock-treated (-) or treated with 30 nM AUY922 for 4 h ( +). Cell lysates were prepared and IP with anti-HSP90 conjugated agarose. Mouse IgG antibodies were served as an IP control. Quantification was performed for three independent experiments. Data are presented as the mean ± s.e.m. The P values were determined by two-tailed Student’s t-test, ** P < 0.01; *** P < 0.001; n.s. = not significant. n = 3. f Quantification analysis, related to Fig. 4e, was performed for three independent experiments. Data are presented as the mean ± s.e.m. The P values were determined by two-tailed Student’s t-test, ** P < 0.01; n.s. = not significant. n = 3. [file 12929_2021_751_MOESM4_ESM.pdf]

**Figure S5**

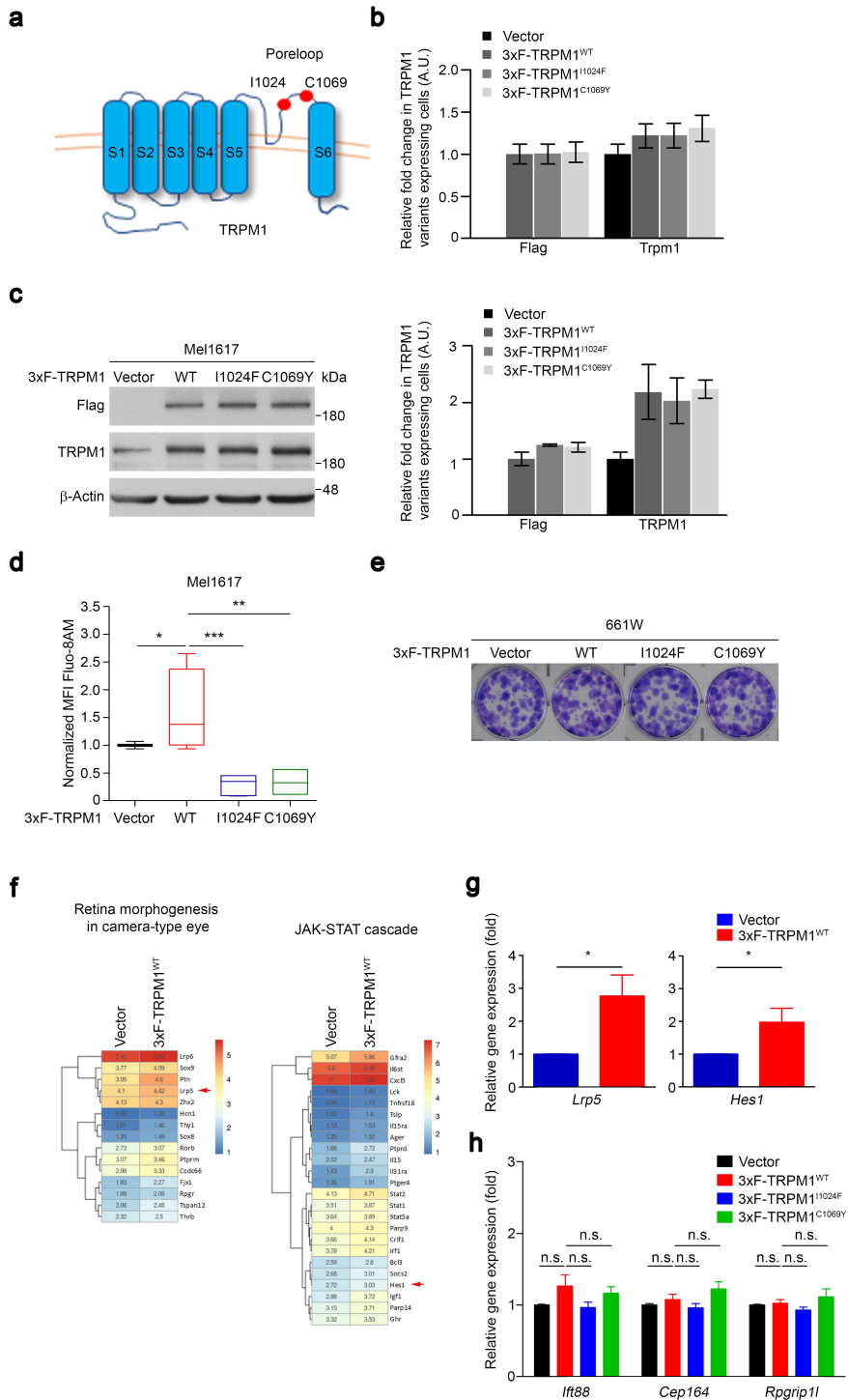

Supplement: Supplementary file 5 — Additional file 5: Figure S5. a Schematic representation of TRPM1 protein. b Quantification analysis, related to Fig. 5a, was performed for three independent experiments. Data are presented as the mean ± s.e.m, n = 3. c Representative western blot results of cells stably expressing either an empty vector or a vector encoding 3xFLAG-tagged wild-type TRPM1 (WT), TRPM1Ile1024Phe (I1024F) or TRPM1Cys1069Tyr(C1069Y). β-Actin was used as a loading control. Quantification was performed for three independent experiments. Data are presented as the mean ± s.e.m. n = 3. d Cytosolic Ca2+ levels were determined by flow cytometry and expressed as the MFI of the Ca2+ dye Fluo-8 AM in cells expressing either an empty vector or variants of TRPM1. Data are presented as the mean ± s.e.m. The P values were determined by two-tailed Student’s t-test, * P < 0.05; ** P < 0.01; *** P < 0.001. n = 3. e Representative images of clonogenic growth assays of 661 W cells expressing an empty vector or variants of TRPM1. Five hundred cells/well were seeded in 6-well plates for 10 d. n = 3. f Heatmaps of gene related to retinal morphogenesis in camera-type eyes (left) and JAK-STAT cascade (right) comparing TRPM1 overexpressing 661 W cells with control cells. g mRNA levels of Lrp5 and Hes1 were determined in cells expressing either an empty vector or variants of TRPM1 by RT-qPCR. mRNA levels were calculated relative to those in cells expressing an empty vector. Levels of the housekeeping gene β-Actin were used as a reference. Data are presented as the mean ± s.e.m. The P values were determined by two-tailed Student’s t-test, * P < 0.05. n = 3. h mRNA levels of lft88, Cep164 and Rpgrip1l were determined in cells expressing either an empty vector or variants of TRPM1 by RT-qPCR. mRNA levels were calculated relative to those in cells expressing an empty vector. Levels of the housekeeping gene β-Actin were used as a reference. Data are presented as the mean ± s.e.m. The P values were determined by two-ta [file 12929_2021_751_MOESM5_ESM.pdf]

**Figure S6****a**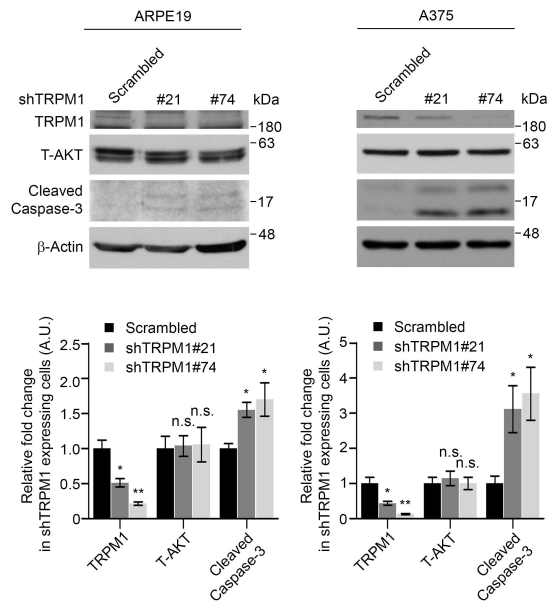**c**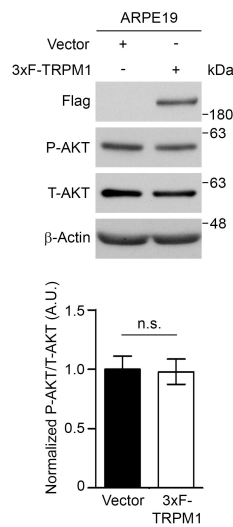**b**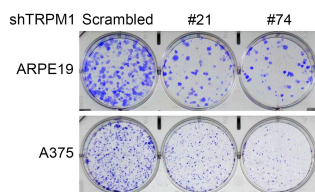**d**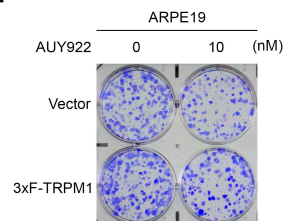**e**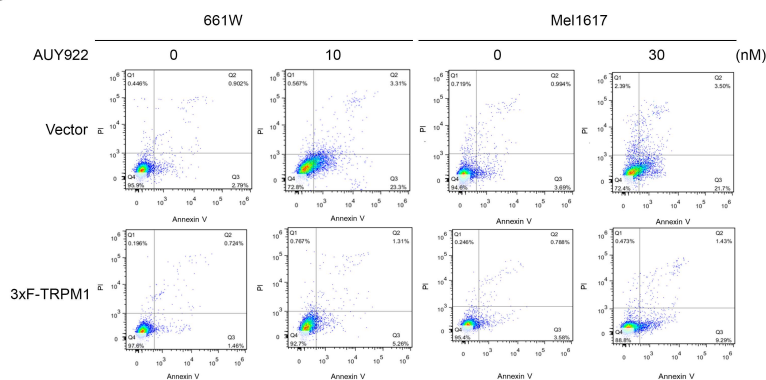**f**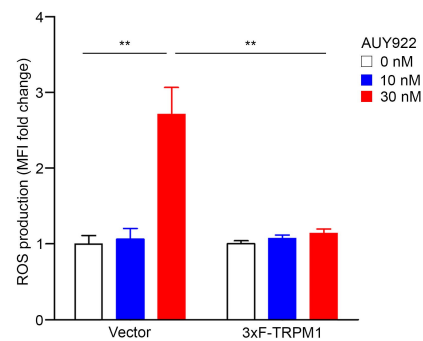

Supplement: Supplementary file 6 — Additional file 6: Figure S6. a Representative western blot results of ARPE19 (left) and A375 (right) cells expressing either scrambled shRNA or shRNAs specific for TRPM1. Quantification analysis was performed for three independent experiments. Data are presented as the mean ± s.e.m. The P values were determined by two-tailed Student’s t-test, * P < 0.05; ** P < 0.01; n.s. = not significant. n = 3. b Representative images of clonogenic growth assays in TRPM1-knockdown cells described in Figure S6a. Five hundred ARPE19 (top) and 3000 A375 (bottom) cells were seeded in 6-well plates for 10 d. n = 3. c Representative western blot results of ARPE19 cells expressing either an empty vector or 3xF-TRPM1. Quantification analysis was performed for three independent experiments. Data are presented as the mean ± s.e.m. Significances were determined by two tailed Student’s t-test, n.s. = not significant. n = 3. d Representative images of clonogenic growth assays in ARPE19 cells stably expressing an empty vector or 3xF-TRPM1 after treatment with the indicated concentrations of AUY922 for 10 d. Five hundred cells/well were seeded in 6-well plates. n = 3. e Representative flow cytometry plots for apoptosis analyses are shown. Cells stably expressing an empty vector or 3xF-TRPM1 were treated with the indicated concentrations of AUY922 for 24 h before apoptosis analyses were performed. n = 3. f ROS levels were measured in 661 W cells treated with AUY922 for 48 h. Cells were labeled with DCFDA (20 μM) and analyzed by flow cytometry. ROS levels are expressed as fold change. Data are presented as the mean ± s.e.m. The P values were determined by two-tailed Student’s t-test, ** P < 0.01. n = 3. [file 12929_2021_751_MOESM6_ESM.pdf]

**Figure S7**

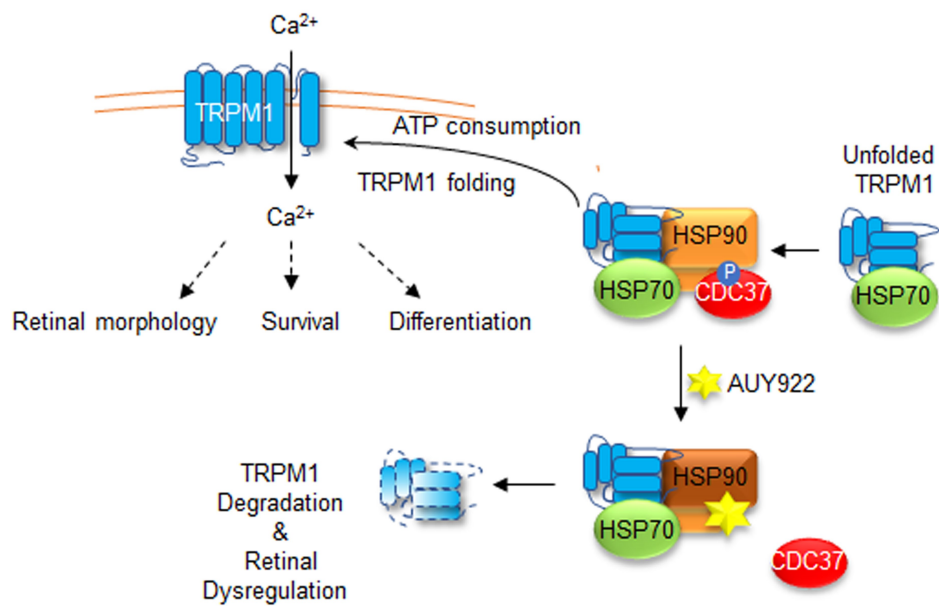

Supplement: Supplementary file 7 — Additional file 7: Figure S7. A schematic representation of the mechanism of AUY922-induced retinal toxicity through attenuation of TRPM1. [file 12929_2021_751_MOESM7_ESM.pdf]
